# Screening and characterization of pro-angiogenic peptides from *Spirulina*: computer simulation, experimental validation, and mechanistic insights

**DOI:** 10.3389/fphar.2026.1885188

**Published:** 2026-08-25

**Authors:** Fenghua Xu, Bing Li, Shanshan Zhang, Yun Zhang, Guohai Su, Yan Zheng

**Affiliations:** 1 Research Center of Translational Medicine, Jinan Central Hospital Affiliated to Shandong First Medical University, Jinan, Shandong Province, China; 2 Department of Genetics and Cell Biology, Basic Medical College, Qingdao University, Qingdao, Shandong Province, China; 3 Biology Institute, Qilu University of Technology (Shandong Academy of Sciences), Jinan, Shandong Province, China; 4 Department of Cardiology, Jinan Central Hospital Affiliated to Shandong First Medical University, Jinan, Shandong Province, China; 5 Shandong Provincial Key Medical and Health Discipline of Jinan Central Hospital, Jinan, China

**Keywords:** angiogenesis, PI3K-akt pathway, pro-angiogenic peptides, virtual screening, zebrafish

## Abstract

In this study, we identified novel pro-angiogenic peptides from *Spirulina* and elucidated their molecular mechanisms. Among the three fractions (A, B, and C) purified from the *Spirulina* protein extract by RP-HPLC, fraction B displayed the most potent pro-angiogenic activity. Through LC-MS/MS analysis, bioinformatics screening, and molecular docking, 20 candidate peptides were selected. In zebrafish models, five of these peptides, MFDCLR, MDMWGK, MNPGCR, MYDNFCK, and MPGLGR, significantly stimulated subintestinal vessel plexus (SIVP) growth and rescued intersegmental vessel (ISV) injury induced by Vatalanib (PTK787). Cellular experiments further showed that the five peptides reversed VEGFR tyrosine kinase inhibitor II (VRI)-induced impairments in proliferation, migration, and tube formation, with MDMWGK exhibiting the most pronounced pro-angiogenic effect. Moreover, the Matrigel plug assay confirmed that MDMWGK promoted extensive neovascularization *in vivo*. Subsequent mechanistic investigations revealed that the pro-angiogenic effect of MDMWGK was associated with activation of the VEGFA/PI3K-Akt signaling pathway, as evidenced by increased phosphorylation of PI3K and Akt. Collectively, this study provides an integrated screening strategy for discovering bioactive peptides from *Spirulina* and establishes a theoretical basis for developing pro-angiogenic agents.

## 1 Introduction

Angiogenesis is a complex, multistep process that results in the formation of new blood vessels (Chen et al., 2024). This process is initiated by the activation of endothelial cells and proteases, which leads to extracellular matrix degradation, followed by endothelial cell migration, proliferation, and differentiation into capillaries (Li et al., 2015). Under normal physiological conditions, a balance between pro-angiogenic and anti-angiogenic factors is essential (Sanchez-Madrid and Sessa, 2010). Insufficient angiogenesis and impaired blood flow are associated with cardiovascular and peripheral artery diseases, including ischemic stroke, atherosclerosis, and hypertension (Carmeliet, 2003). However, safe and effective pro-angiogenic drugs for treating these conditions remain unavailable in clinical practice. Therefore, the identification of highly effective therapeutic agents that promote angiogenesis represents a critical strategy for treating ischemic diseases. Although numerous studies have screened and identified compounds with specific angiogenic effects, the majority are anti-angiogenic agents targeting angiogenesis-dependent diseases. In contrast, research on pro-angiogenic compounds for disorders linked to insufficient angiogenesis remains limited. Consequently, the discovery of highly effective and low-toxic pro-angiogenic compounds is of particular importance for developing therapies for these disorders.

The ocean is a rich source of natural medicinal compounds, and the search for highly effective, low-toxicity drugs from marine sources has garnered increasing interest (Xing et al., 2022). Marine natural products have emerged as important resources for novel drugs, functional foods, biomaterials, and cosmetics (Vladkova et al., 2022; Wu et al., 2025). Among marine-derived bioactive substances, peptides are particularly attractive due to their favorable safety profiles, low toxicity, and cost-effective production, and have become promising candidates for pharmaceutical and functional food development (Xing et al., 2022; Cao et al., 2026). Numerous studies have demonstrated that peptides derived from marine algae, such as *Spirulina*, kelp, and Sargassum, possess immunomodulatory, anti-diabetic, anti-tumor, neuroprotective, anti-inflammatory, and cardioprotective activities (Ghallab et al., 2024; Meng et al., 2025; Wang et al., 2025). For example, enzymatic hydrolysis of a *Spirulina protein extract* releases bioactive peptides; in our previous study on the hydrolysates of this extract, three fractions (A, B, and C) were isolated, among which fraction C exhibited remarkable antioxidant activity (Xu et al., 2022). Notably, fraction B demonstrated significant pro-angiogenic activity (Supplementary Figure S1). This unexpected finding prompted us to further investigate the peptide constituents responsible for this effect.

Molecular docking, a core technique in computer-aided drug design, simulates receptor–ligand interactions and has become a powerful tool for identifying active compounds from large chemical libraries (Rampogu et al., 2020). For example, Fan et al. (2024) successfully used molecular docking to reveal the potential mechanisms of 10 novel anti-inflammatory peptides from duck liver. The results suggested that these peptides may exert anti-inflammatory effects by binding to key inflammatory protein pockets and inhibiting the NF-κB pathway, thereby highlighting the potential of this technique for screening natural bioactive peptides. Chen et al. (2022) isolated fresh-tasting peptides from mushrooms and identified the sites of action of the peptides with the fresh-tasting receptor T1R1/T1R3 by molecular docking. Majid et al. (2022) identified DPP4 inhibitory peptides in sorghum using molecular docking combined with *in vitro* and Caco-2 cell *in situ* assays. Additionally, antioxidant and anti-inflammatory peptides from *Ulva prolifera* have been investigated using molecular docking (Liu et al., 2025). In addition to molecular docking, bioinformatics tools such as PeptideRanker and the BIOPEP database serve as complementary approaches for predicting peptide bioactivity prior to experimental validation (Cao et al., 2026). This pre-selection process helps optimize peptide design and improve research efficiency.

In this study, we aimed to identify pro-angiogenic peptides from a *Spirulina* protein extract and to elucidate their molecular mechanisms. To this end, we employed an integrated strategy combining RP-HPLC fractionation, LC-MS/MS identification, bioinformatics prediction, molecular docking, and zebrafish-based functional screening. Sequence alignment against the Arthrospira platensis proteome confirmed that the identified peptides originated from Spirulina proteins. Using HUVEC models, a Matrigel plug assay, and transcriptomic analysis, we further demonstrated that MDMWGK promotes angiogenesis through activation of the VEGFA/PI3K-Akt signaling pathway. Collectively, this study provides an integrated screening strategy for discovering bioactive peptides from algal protein extracts and establishes a theoretical basis for developing food-derived pro-angiogenic agents for ischemic diseases.

## 2 Materials and methods

### 2.1 Material and reagents

A reagent-grade *Spirulina* protein extract (Binmei Biotechnology, Taizhou, China) was used as the raw material for enzymatic hydrolysis. Fractions A, B, and C, previously isolated and purified from the hydrolysates of this extract by reversed-phase high-performance liquid chromatography (RP-HPLC) in our previous study (Xu et al., 2022), were used for subsequent activity evaluation. All 20 synthetic peptides (MAYFWFK, MSYFWK, MFDCLR, MDMWGK, MPLAPWR, MNPGCR, MFFWEK, MQFPNR, MFDAFTR, MYDNFCK, MPSFDPR, MPECPVCR, MPGLGR, MFAKPR, MICPNR, MRFSFPK, MSPFYK, MNPWQGK, MGFYSR, and MPWAGAGR) with a purity of ≥95% were synthesized by Cellmano Biotech Ltd (Hefei, China). Tricaine was obtained from Sigma-Aldrich (St. Louis, MO, USA). Vatalanib (PTK787) was obtained from Sigma-Aldrich (Shanghai, China). The VEGFR tyrosine kinase inhibitor II (VRI) was purchased from Merck KGaA (Darmstadt, Germany). Recombinant Human VEGF165 protein was purchased from Novoprotein Scientific Inc. (Suzhou, China). Human umbilical vein endothelial cells (HUVECs) were purchased from Wuhan Pricella Biotechnology Co., Ltd (Wuhan, China). Human umbilical vein endothelial cell complete culture medium (containing 5% (v/v) fetal bovine serum, 1% (v/v) endothelial cell growth supplement, 100 U/mL penicillin, and 100 μg/mL streptomycin) was purchased from ScienCell Research Laboratories (Carlsbad, CA, USA). Matrigel® was obtained from Corning (Corning, NY, USA). Phosphate-buffered saline (PBS), trypsin-EDTA solution, and gelatin solution were purchased from Solarbio Science and Technology Co., Ltd (Beijing, China). The Cell Counting Kit-8 (CCK-8) and BCA Protein Assay Kit were provided by Beyotime Biotechnology Co., Ltd (Shanghai, China). The Human VEGF Quantikine ELISA Kit was acquired from R&D Systems (Minneapolis, MN, USA). The Tissue Total RNA Isolation Kit was purchased from Vazyme Biotech Co., Ltd (Nanjing, China). All other chemicals used during experiment were of analytical grade.

### 2.2 Zebrafish maintenance

The transgenic zebrafish line Tg (fli1a: EGFP), which expresses enhanced green fluorescent protein in the vasculature, was obtained from the Engineering Research Center of Zebrafish Models for Human Diseases and Drug Screening in Shandong Province (Jinan, China). Zebrafish were maintained under standard laboratory conditions with a cycle of 14 h of light and 10 h of dark as previously described (Xu et al., 2023; Xu et al., 2024). Embryos were obtained through natural spawning of adult zebrafish at a 1:1 male-to-female ratio, and collected between 9:00 and 10:00 a.m. the next day. The embryos were then cultured in embryo water (5 mM NaCl, 0.17 mM KCl, 0.4 mM CaCl_2_, and 0.16 mM MgSO_4_) at 28 °C ± 0.5 °C in an incubator.

### 2.3 Pro-angiogenic activity of fractions in zebrafish

The pro-angiogenic activities of fractions A, B, and C, obtained from our previous study (Xu et al., 2022), were evaluated using the transgenic Tg (fli1a:EGFP) zebrafish model. At 20 h post fertilization (hpf), embryos were dechorionated using 1 mg/mL Pronase E. Healthy embryos were randomly transferred into 24-well plates with 10 embryos per well. All wells were incubated with 30 μg/mL N-phenylthiourea to inhibit melanin production. The embryos were then divided into the following groups: a control group treated with 0.1% DMSO (v/v), a model group treated with 0.1 μg/mL PTK787 (Vatalanib), and three experimental groups treated with 0.1 μg/mL PTK787 and 100 μg/mL of fraction A, fraction B, or fraction C, respectively. Each group was tested in triplicate. Embryos were incubated in a light-controlled incubator at a constant temperature of 28 °C for 24 h. Subsequently, the larvae were anesthetized with 0.02% tricaine methanesulfonate, and images of the intersegmental vessels (ISVs) in the trunk and tail regions were captured using a fluorescence microscope (Olympus IX53, Japan). The experiment was repeated three times independently.

In addition, zebrafish larvae were treated at 72 hpf with fractions A, B, and C. After 24 h, images were acquired using a fluorescence microscope (AXIO-V16, Zeiss, Germany). The total length of the subintestinal vessel plexus (SIVP) was measured using image processing software. Based on this, the fraction with the highest pro-angiogenic activity was selected for subsequent peptide identification.

### 2.4 Peptide identification

Fraction B, which exhibited the greatest pro-angiogenic activity, was subjected to liquid chromatography-tandem mass spectrometry (LC-MS/MS) for peptide sequence identification. Briefly, mobile phase A (100% water, 0.1% formic acid) and B solution (80% acetonitrile, 0.1% formic acid) were prepared. The fraction B was dissolved in 10 μL of solution A, centrifuged at 14,000 *g* for 20 min at 4 °C, and 1 μg of the supernatant was injected into a home-made C18 Nano-Trap column (4.5 cm × 75 μm, 3 μm). A homemade analytical column (25 cm × 150 μm, 1.9 μm) was used, the column oven was set as 55 °C. The separated peptides were analyzed by Orbitrap Exploris 480 matched with FAIMS (Thermo Fisher), with ion source of Nanospray Flex™ (ESI), spray voltage of 2.1 kV and ion transport capillary temperature of 320 °C. Data-dependent acquisition mode was adopted by mass spectrometry, FAIMS compensation voltage was set as −45 and −65 respectively, and followed to the same acquisition parameters: full scan range from m/z 350 to 1500 with resolution of 60,000 (at *m/z* 200), an automatic gain control (AGC) target value was 3 × 10^6^ and a maximum ion injection time was Auto. The scan-round time in MS/MS was set to 1s, and the precursors in the full scan were selected from high to low abundance and fragmented by higher energy collisional dissociation (HCD), where resolution was 15,000 (at *m/z* 200), the automatic gain control (AGC) target value was 7.5 × 10^4^, the maximum ion injection time was 22 m, a normalized collision energy was set as 30%, an intensity threshold was 5.0 × 10^3^, and the dynamic exclusion parameter was 40 s.

The MS/MS spectra of the 20 candidate peptides are presented in Supplementary Figure S2. To trace the protein origins of these peptides, we performed sequence alignment against the Arthrospira platensis proteome (UniProt); the identification results, including peptide sequences, UniProt accession numbers, protein names, and precursor protein sequences, are summarized in Supplementary Table S1.

### 2.5 Sequence-based bioactivity prediction

The PeptideRanker (http://distilldeep.ucd.ie/PeptideRanker/) was used to predict the activity of the peptides. Peptides with an activity score greater than 0.7 were retained. In addition, all of the peptides were checked for toxicity using the ToxinPred server (http://crdd.osdd.net/raghava/toxinpred/) (Gupta et al., 2013). Finally, the hydrophobicity, net charge, and isoelectric point (pI) of these peptide sequences were calculated and predicted by the PepDraw tool (http://pepdraw.com/).

### 2.6 Molecular docking

The 3D structures of the target receptors, including epidermal growth factor receptor (EGFR) tyrosine kinase domain (PDB ID: 1M17), fibroblast growth factor receptor 1 (FGFR1) extracellular domain (PDB ID: 3C4F), vascular endothelial growth factor receptor 1 (VEGFR1; PDB ID: 3HNG), vascular endothelial growth factor receptor 2 (VEGFR2; PDB ID: 3WZE), and protein kinase B (PKB/Akt1; PDB ID: 3OW4), were retrieved from the RCSB Protein Data Bank (RCSB PDB; https://www.rcsb.org/). The 3D structures of 41 potential candidate peptides were constructed using ChemDraw and Chem3D software. In addition, the peptides were energy-minimized using the Macromolecular module and the Chemistry at Harvard Macromolecular Mechanics (CHARMm) forcefield in Discovery Studio 2020 to obtain the ligand molecules (Lu et al., 2025). Then, the binding receptor was pretreated by removing water molecules and small molecule ligands from the receptors as well as repairing incomplete side chains using the Clean Protein program. Finally, molecular docking was performed using the LibDock module with default parameters. A heatmap of the LibDock scores was constructed using GraphPad Prism Software. To identify peptides with balanced binding affinities across multiple angiogenesis-related targets rather than those that bind favorably to only a single receptor, the average LibDock score across the five targets was used as the primary ranking metric. Accordingly, the top 20 peptides with the highest average LibDock scores across the five targets were selected as candidate peptides for subsequent experimental validation.

### 2.7 Peptide synthesis

Selected peptides were synthesized by Cellmano Biotech (Hefei) Co., Ltd (China) using the standard Fmoc solid-phase synthesis strategy. The peptides were synthesized with free N-termini (NH_2_) and free C-termini (COOH), without acetylation or amidation modifications. The purity of each synthetic peptide was confirmed to be over 95% by analytical reversed-phase high performance liquid chromatography-tandem mass spectrometry (RP-HPLC-MS/MS), and the chromatograms for the five representative peptides are shown in Supplementary Figure S3. Lyophilized peptides were stored at −20 °C and dissolved in Milli-Q water to a desired concentration before use.

### 2.8 Pro-angiogenic activity of peptides in zebrafish

To validate the pro-angiogenic activity of the candidate peptides identified by molecular docking, the 20 synthesized peptides were evaluated using the PTK787-induced zebrafish model as described in Section 2.3. Briefly, 24 hpf zebrafish embryos were divided into multiple groups: a normal control group (0.1% DMSO), a model group (0.1 μg/mL PTK787), a positive control group (0.1 μg/mL PTK787 + 100 ng/mL VEGF), and treatment groups co-treated with 0.1 μg/mL PTK787 and each of the 20 synthetic peptides at a concentration of 10 μg/mL. After 24 h of incubation at 28 °C, the zebrafish larvae were anesthetized, and the formation of ISVs was observed and quantified as described in Section 2.3.

For SIVP assay, 72 hpf larvae were treated with peptides for 24 h, anesthetized, and imaged. SIVP length was quantified by imaging software.

### 2.9 Cell viability assay

HUVECs were cultured at 37 °C in complete endothelial cell medium under a humidified atmosphere of 5% CO_2_ and 95% air. The cells were seeded into 96-well plates at a density of 2 × 10^4^ cells per well and allowed to adhere for 24 h. Subsequently, they were treated separately with the peptides MFDCLR, MDMWGK, MNPGCR, MYDNFCK, and MPGLGR for 24 h. The culture medium was then replaced with 100 μL of medium containing 10% CCK-8 solution, and the cells were incubated for 1 h to assess viability. The absorbance at 450 nm wavelength was measured by SpectraMax M5 (Wallac, Netherlands).

Cell viability assays under VRI were performed using the modified method of Liu et al. (2023). Cells were seeded at 2 × 10^4^ cells/well into 96-well microplates. After 24 h of incubation, the model group was treated with 50 μM VRI. The positive control group was treated with 50 μM VRI and 40 ng/mL VEGF. The peptide treatment groups were treated with 50 μM VRI and each peptide. After 24 h, 10 μL CCK-8 was added to the cells and incubated for 1 h. Thereafter, the absorbance was measured at 450 nm using a microplate reader.

### 2.10 HUVECs wound healing assay

HUVECs were seeded in 24-well plates at a density of 2 × 10^5^ cells per well and cultured under standard conditions for 24 h until they reached 90% confluency. Cell migration was assessed by creating a scratch wound across the center of each well using a 200 μL pipette tip. After washing with PBS to remove detached cells, the cells were incubated in fresh medium containing 10% FBS, with or without peptide treatment. Images of each well were captured under an inverted microscope at 0, 6, and 12 h. The cell migration rate was analyzed using ImageJ software.

### 2.11 HUVECs tube formation assay

Matrigel assay was performed to evaluate *in vitro* angiogenic activity via quantifying tube formation as previously described (Chen et al., 2024). Briefly, after overnight thawing at 4 °C, 100 μL of diluted Matrigel was added into each well of 96-well plate and incubated for 30 min at 37 °C for solidification. HUVECs were seeded on the Matrigel at a concentration of 4 × 10^4^ cells/well with different treatments. The cells were subsequently incubated at 37 °C with 5% CO_2_ for 6 h. Subsequently, tube structures were observed and imaged using an Olympus inverted microscope. The length of the tubes was quantified using ImageJ software.

### 2.12 ELISA kit measurement

HUVECs were rinsed twice with pre-cooled PBS and then digested with 0.25% trypsin-EDTA solution. After centrifugation at 1500 rpm for 15 min, the supernatant was collected for subsequent detection. The concentration of vascular endothelial growth factor (VEGF) in the supernatant was determined using a commercial VEGF ELISA kit, strictly following the manufacturer’s protocol.

### 2.13 Quantitative real-time RT-PCR

Total RNA was extracted from HUVECs in each group using the FastPure Cell/Tissue Total RNA Isolation Kit V2 (Vazyme, Nanjing, China) and reverse-transcribed with HiScript II Q RT SuperMix for qPCR (Vazyme). Quantitative real-time PCR (qPCR) was then performed on a LightCycler® 96 System (Roche, Switzerland) using TransScript® Green One-Step qRT-PCR SuperMix (TIANGEN Biotech). The amplification conditions were 94 °C for 30 s, followed by 40 cycles of 94 °C for 5 s, 60 °C for 15 s, and 72 °C for 10 s. The primer sequences used are listed in Supplementary Tables S2 and S3. GAPDH was used as the internal control, and the relative gene expression was calculated using the 2^−ΔΔCT^ method.

### 2.14 *In vivo* Matrigel plug assay

Animal experiments were approved by the Animal Care Committee of Jinan Central Hospital Affiliated to Shandong First Medical University. For Matrigel plug assay, mice were anesthetized with 1.5%–2% isoflurane vaporized in oxygen. After 14 days, the mice were euthanized using an overdose of anesthesia with 1%–1.5% isoflurane, followed by removal of the plugs for further analysis.

Briefly, a mixture containing 500 μL Matrigel, 50 ng/mL VEGF, and 30 U/mL heparin, alone or with the MDMWGK peptide, was subcutaneously injected into the dorsal surface of the mice, leading to the formation of a solid plug. Mice were euthanized after 14 days, and the Matrigel plugs were removed, photographed, and fixed in 4% paraformaldehyde. Staining with the Cluster of Differentiation 31 (CD31) antibody was used for qualitative identification of endothelial cells (ECs) in the histological tissue sections.

### 2.15 Transcriptome assay

HUVECs were rinsed twice with PBS and treated with RNase-free DNase I (Takara, Kusatsu, Japan). RNA was quantified using Agilent 2100 Bioanalyzer (Agilent Technologies, CA, USA), the quality and integrity were assessed by NanoDrop spectrophotometer (Thermo Scientific, DE, USA). The libraries were constructed using the TruSeq Stranded mRNA LT Sample Prep Kit (Illumina, San Diego, CA, USA) following the manufacturer’s instructions. Then, transcriptome sequencing and analysis were conducted by OE Biotech Co., Ltd (Shanghai, China). In this study, analysis of differentially expressed genes was performed as follows: the VRI-treated and control groups were compared (model vs. control), and the peptide MDMWGK-treated and VRI-treated groups were compared (MDMWGK vs. model). Genes with significantly differential expression, as defined by a P value <0.05 and fold change ≥1.5, were selected for subsequent analysis. Then, the expression patterns of differentially expressed genes (DEGs) in different groups were revealed by hierarchical cluster analysis. These DEGs were used for Gene Ontology (GO) enrichment and Kyoto Encyclopedia of Genes and Genomes (KEGG) pathway enrichment analysis using R based on the hypergeometric distribution.

### 2.16 Western blot analysis

HUVECs were washed twice with PBS and collected. The total proteins for the western blot were extracted by RIPA buffer with protease inhibitor cocktail and measured by a BCA assay. Protein samples were separated on 10% SDS-PAGE gels and then transferred to PVDF membranes. Membranes were blocked with 5% non-fat dry milk at roomtemperature for 1 h, followed by overnight incubation with primary antibodies VEGFA (1:1000, 19003-1-AP, Proteintech, US), PI3K (1:1000, 4292S, CST, US), p-PI3K (1:1000, 4228T, CST, US), Akt (1:1000, 9272S, CST, US), p-Akt (1:1000, 9271S, CST, US), p38 (1:1000, 9212S, CST, US), p-p38 (1:1000, 9211S, CST, US) at 4 °C. Subsequently, an electrochemiluminescence chromogenic substrate was added, and the target bands were visualized using the automatic chemiluminescence image analysis system (5200 Multi, Tanon, China). The band of GAPDH was used as an internal standard to normalize the results and analyzed by ImageJ software.

### 2.17 Statistical analysis

All results are expressed as the mean ± standard deviation, as indicated. Statistical analyses were performed using GraphPad Prism Software. A one-way analysis of variance (ANOVA) with a Tukey’s post hoc test was used to evaluate statistically significant differences in data from multiple groups (*P* > 0.05 (n.s.), ^*^ *P* < 0.05, ^**^ *P* < 0.01). Statistical significance between two groups was evaluated using a Student’s t-test (*P* > 0.05 (n.s.), ^*^ *P* < 0.05, ^**^ *P* < 0.01) (n.s., not significant).

## 3 Results

### 3.1 Screening of potential bioactive peptides

A total of 114 peptide sequences were identified from fraction B by LC-MS/MS. These peptides ranged from 6 to 10 amino acids, with molecular weights between 600 and 1300 Da. To screen for bioactive candidates, we predicted peptide activity using PeptideRanker, which identified 41 peptides with activity scores >0.7. The physicochemical properties of these peptides are summarized in Table 1. Toxicity prediction of the potential bioactive peptides using the online tool *ToxinPred* with SVM >0.5 setting showed that none of the peptides had potential toxic effects. However, *in silico* tools have certain limitations (Rajendran et al., 2016) and evaluation of the *in vivo* toxicity and bioactivity would require future experimental validation.

**TABLE 1:** Physicochemical properties and predicted bioactivity of 41 peptides identified and characterized from *Spirulina*.

Peptide sequence	Amino acid numbers	Peptide mass (Da)	PeptideRanker score	Hydrophobicity (kJ/mol)	Net electric charge	Isoelectric point (pI)	Instability coefficient	Aliphatic index
MAYFWFK	7	992.19	0.976,349	+4.31	+1	9.58	4.73	14.29
MFGFFNR	7	918.07	0.964,697	+5.98	+1	9.58	81.57	0.00
MSVPGWFWR	9	1165.37	0.964,061	+9.70	0	6.07	93.10	65.00
MFMFKR	6	859.12	0.94788	+9.02	+2	11.53	25.31	0.00
MNQWLWR	7	1033.21	0.928,623	+12.06	0	6.54	−23.10	0.00
MPLAPWR	7	870.08	0.926,788	+8.80	+1	10.88	146.74	55.71
MSYFWK	6	861.02	0.926,631	+11.16	+1	8.79	3.53	0.00
MFFWEK	6	887.06	0.904,423	+8.21	+1	9.58	241.23	0.00
MFDCLR	6	783.96	0.90147	+8.38	+1	10.88	87.75	48.75
MRFSFPK	7	912.11	0.881,601	+9.09	+1	10.88	15.30	0.00
MSVFGFR	7	843.01	0.881,225	+10.01	0	6.54	26.20	14.29
MPSFDPR	7	848.97	0.870,612	+10.39	+1	10.88	30.67	25.00
MDMWGK	6	766.93	0.853,972	+12.08	0	6.07	54.76	0.00
MPSPIPR	7	796.98	0.849,141	+8.78	+2	12.49	141.86	70.00
MNPGCR	6	676.81	0.844,467	+12.45	0	6.02	188.47	36.25
MSPFYK	6	771.92	0.830,942	+10.85	+1	9.91	−11.79	0.00
MSLPDWFANR	10	1236.4	0.828,414	+10.23	+1	10.88	81.57	65.00
MSNPVFCR	8	953.14	0.823,484	+10.77	+2	11.53	−16.80	16.67
MMNLDFFYGR	10	1293.52	0.814,175	+8.89	+1	8.79	40.43	65.00
MSPNPWSQLR	10	1215.38	0.80282	+8.89	+1	8.79	128.50	48.75
MNSPFLNR	8	978.13	0.795,085	+8.38	+1	10.88	87.75	48.75
MIKGWCR	7	893.14	0.78853	+9.76	+2	9.94	1.94	5.71
MQFPNR	6	791.92	0.779,486	9.09	+1	10.88	15.30	0.00
MISLWPIIR	9	1128.43	0.778,041	2.94	+1	10.88	35.20	173.33
MPFSVLLR	8	962.21	0.77654	4.97	+1	10.88	111.83	133.75
MFDAFTR	7	887.02	0.771,006	10.01	0	6.54	26.20	14.29
MPWAGAGR	8	844.98	0.768,072	10.39	+1	10.88	30.67	25.00
MYDNFCK	7	920.07	0.76792	12.08	0	6.07	54.76	0.00
MDLHFWTPK	9	1174.37	0.760,028	11.34	0	7.65	−19.33	43.33
MDMWGKWAK	9	1152.39	0.756,749	13.27	+1	9.73	−28.77	11.11
MALWQRR	7	960.16	0.740,421	8.78	+2	12.49	141.86	70.00
MPECPVCR	8	934.17	0.739,402	12.45	0	6.02	188.47	36.25
MNPWQGK	7	859.99	0.736,255	10.85	+1	9.91	−11.79	0.00
MNNPVWR	7	916.06	0.734,675	8.33	+1	10.88	31.97	41.43
MPGLGR	6	629.78	0.733,657	10.23	+1	10.88	81.57	65.00
MLGEMWSFR	9	1156.38	0.730,562	8.56	0	6.64	0.51	43.33
MFAKPR	6	748.94	0.725,522	10.77	+2	11.53	−16.80	16.67
MGFYSR	6	759.87	0.710,191	8.23	+1	9.68	94.77	0.00
MICPNR	6	732.92	0.704,254	8.89	+1	8.79	40.43	65.00
MANFCDR	7	855.99	0.70364	12.30	0	6.07	21.47	4.29
MSCPTPIR	8	904.11	0.701,991	8.89	+1	8.79	128.50	48.75

### 3.2 Molecular docking of 41 potential bioactive peptides with receptor proteins

**FIGURE 1:**
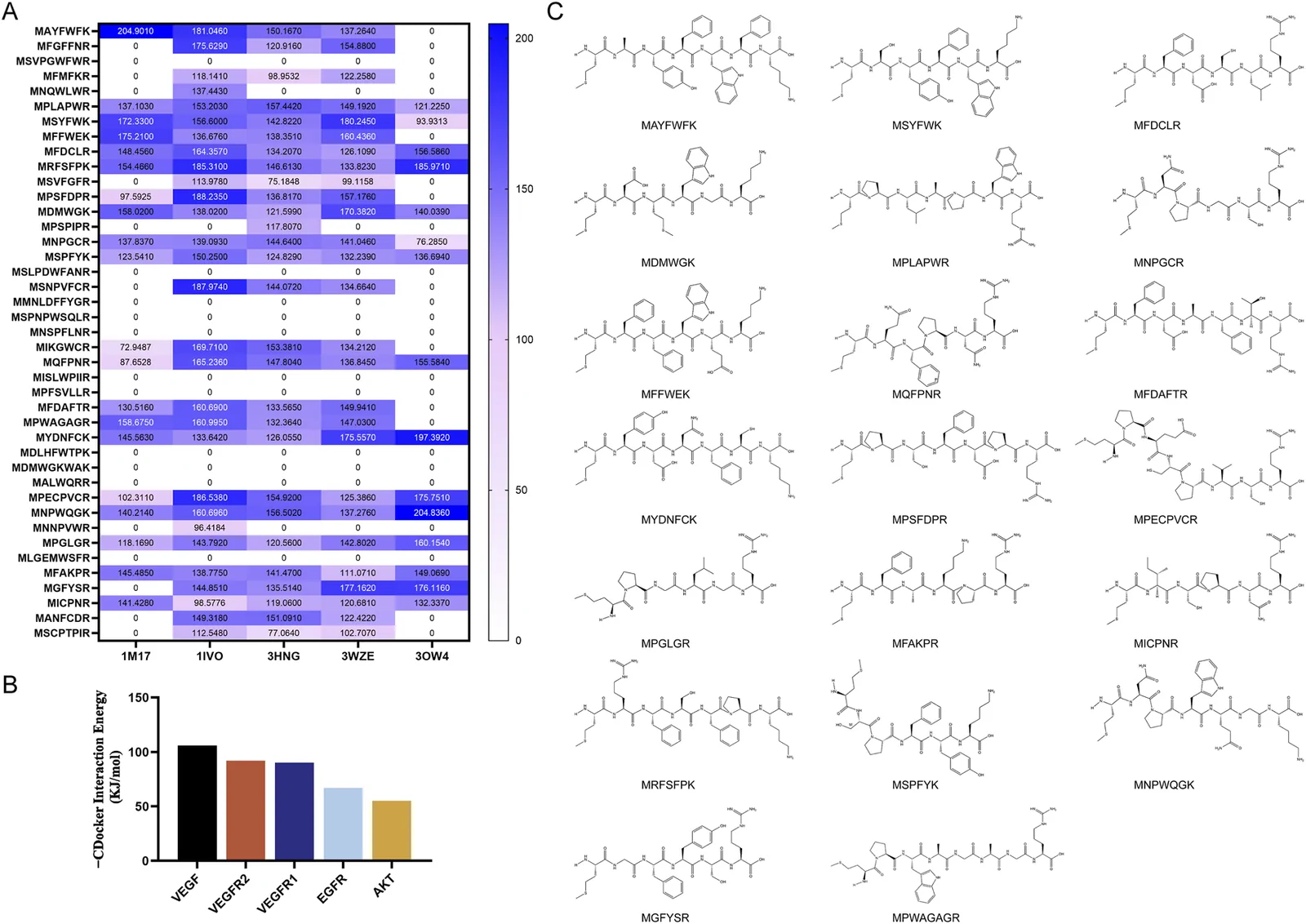
Molecular docking analysis of 41 potential bioactive peptides interactions with angiogenesis-related targets **(A)** LibDock interaction energy heatmap of 41 peptides (columns) against 5 core targets (rows), color-scaled by docking energy (kcal/mol). EGFR (PDB ID: 1M17), FGFR1 (PDB ID: 3C4F), VEGFR1 (PDB ID: 3HNG), VEGFR2 (PDB ID: 3WZE), Akt (PDB ID: 3OW4) **(B)** Ranked average binding energies of 5 targets with 41 peptides. Targets are sorted by mean binding energy **(C)** Chemical structures of the 20 prioritized peptides.

Molecular docking is a powerful tool for studying ligand-receptor interactions and has been widely applied in the discovery and screening of bioactive peptides (Huang et al., 2024). To further prioritize peptides with potential pro-angiogenic activity, we docked the 41 predicted peptides into selected target receptors (EGFR, FGFR1, VEGFR1, VEGFR2, and Akt) using the LibDock module. The binding affinities for these peptides to the receptors were evaluated based on their LibDock scores. Typically, a lower (more negative) LibDock score value indicates a more stable and favorable interaction between the ligand and the protein (Feng et al., 2020). Our results indicated that all peptides showed binding with the active sites of the receptor proteins, with LibDock scores ranging from −204.9 to 0 kcal/mol. To visualize and compare the binding patterns, a heatmap was constructed based on the LibDock scores (Figure 1A). Additionally, the average LibDock scores for the five targets were ranked to illustrate their mean binding affinities with the 41 peptides, as shown in Figure 1B. Subsequently, to identify the most promising candidates, the 20 peptides with the highest average LibDock scores across the five targets were selected for further verification. The chemical structures of the 20 peptides are shown in Figure 1C.

Although molecular docking serves as a valuable tool for the preliminary screening of bioactive peptides, *in silico* predictions possess inherent limitations. Therefore, the potential pro-angiogenic peptides screened and predicted through this computational approach necessitated further confirmation via biological activity evaluation.

### 3.3 Validation of the pro-angiogenic effects of 20 candidate peptides in a zebrafish model

**FIGURE 2:**
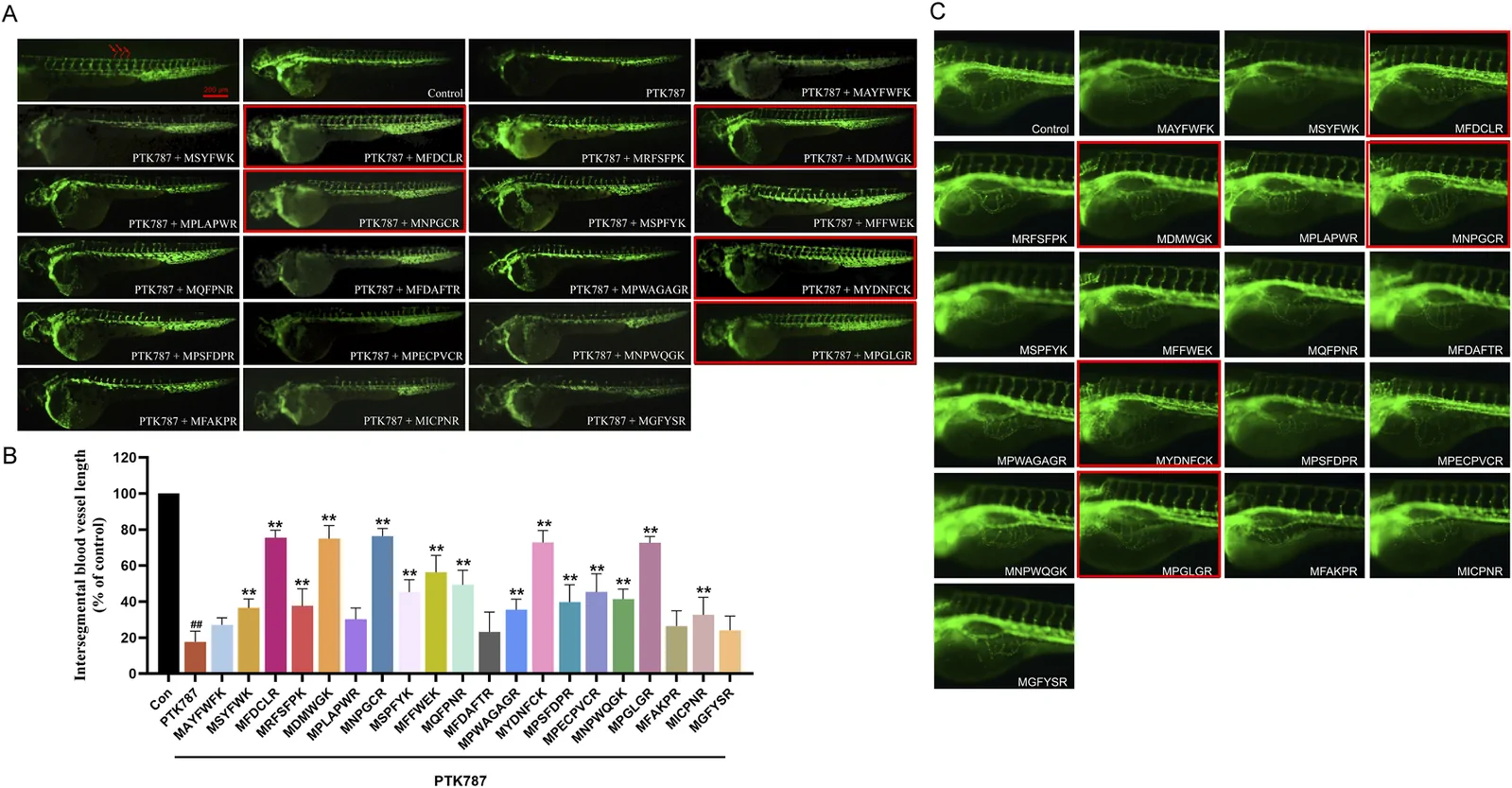
Effects of 20 peptides on intersegmental vessels (ISVs) and subintestinal vessel plexus (SIVP) in zebrafish **(A)** Zebrafish embryos at 24 h post-fertilization (hpf) were treated with PTK787 alone or together with each of the 20 peptides for 24 h. Images were captured at 48 hpf **(B)** Quantitative analysis of the restorative effects of different peptides on PTK787-induced ISV impairment. Values are shown as means ± SD *(*n = 10). ^*##*^ *P* < 0.01 vs. control group. ^**^ *P* < 0.01 vs. model group **(C)** Zebrafish larvae at 72 hpf were treated with different peptides for 24 h. Images were captured at the end of the treatment (96 hpf).

To further validate their pro-angiogenic activity, we synthesized the 20 candidate peptides selected through virtual screening and evaluated them in a zebrafish model. Intersegmental vessels (ISVs) formation, an angiogenic process during zebrafish embryonic development, was used to evaluate the effects of the peptides on angiogenesis (Ding et al., 2025). As shown in Figures 2A,B, Vatalanib (PTK787) treatment significantly inhibited the growth of intact ISVs in zebrafish embryos. In this model, PTK787 was used as a pharmacological tool to create vascular injury, enabling functional screening of the 20 candidate peptides for *in vivo* pro-angiogenic activity. Further evaluation revealed that 15 peptides, including MSYFWK, MFDCLR, MRFSFPK, MDMWGK, MNPGCR, MSPFYK, MFFWEK, MQFPNR, MPWAGAGR, MYDNFCK, MPSFDPR, MPECPVCR, MNPWQGK, MPGLGR, and MICPNR, effectively promoted angiogenesis *in vivo*. Notably, MFDCLR, MDMWGK, MNPGCR, MYDNFCK, and MPGLGR each significantly reversed PTK787-induced vascular defect, and markedly increased the length of subintestinal vessel plexus (SIVP) in zebrafish (Figure 2C). Based on these findings, these five peptides were selected for further characterization in HUVECs.

### 3.4 Effects of five selected peptides on HUVEC viability, migration and tube formation

To further characterize the pro-angiogenic potential of the five candidate peptides selected from the zebrafish screening, we assessed their effects on cell viability, migration, and tube formation using HUVECs. First, a CCK-8 assay was performed to evaluate the effects of these peptides on cell viability. As shown in Supplementary Figure S4A, treatment with each peptide alone significantly increased cell viability compared to the control. In addition, we investigated whether the five peptides protected HUVECs from VEGFR tyrosine kinase inhibitor II (VRI)-induced injury. The results indicated that VRI treatment reduced cell viability, whereas co-treatment with the peptides significantly reversed this inhibitory effect (Supplementary Figure S4B).

**FIGURE 3:**
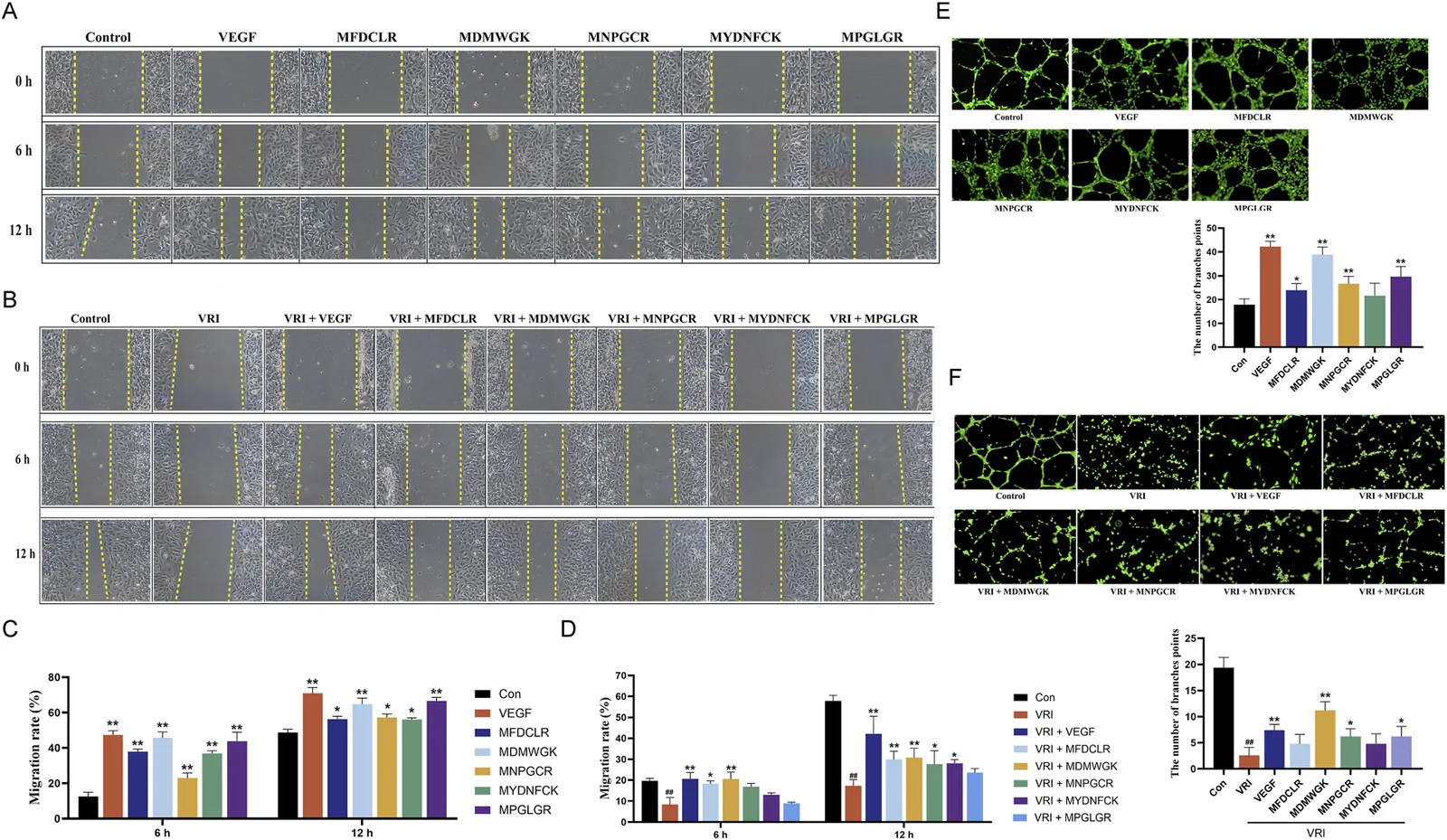
Effects of five selected peptides on HUVEC migration and tube formation **(A)** Microscopic images of cell migration at 0 h, 6 h, and 12 h after treatment with different peptides **(B)** Microscopic images of cell migration at 0 h, 6 h, and 12 h after treatment with VRI and different peptides **(C)** Quantitative analysis of cell migration shown in **(A)**. Values are shown as means ± SD (n = 3). ^*^ *P* < 0.05, ^**^ *P* < 0.01 vs. Control group **(D)** Quantitative analysis of cell migration shown in **(B)**. Values are shown as means ± SD (n = 3). ^*##*^ *P* < 0.01 vs. Control group, ^*^ *P* < 0.05, ^**^ *P* < 0.01 vs. VRI group **(E)** Representative images and quantification of tube branch number in Matrigel tube formation assay after treatment with different peptides. Values are shown as means ± SD (n = 3). ^*^ *P* < 0.05, ^**^ *P* < 0.01 vs. Control group **(F)** Representative images and quantification of tube branch number in Matrigel tube formation assay after treatment with VRI and different peptides. Values are shown as means ± SD (n = 3). ^*##*^ *P* < 0.01 vs. Control group, ^*^ *P* < 0.05, ^**^ *P* < 0.01 vs. VRI group.

Angiogenesis is a complex process in which endothelial cell migration and tube formation are important steps (Wang et al., 2024). The effect of the five peptides on the migration of endothelial cells was assessed using the scratch wound healing assay. As shown in Figures 3A,C, after treatment with VEGF or each of the five peptides for 6 h and 12 h, the scratch width of HUVEC monolayers was significantly smaller than that of the control group, indicating a marked increase in cell migration toward the wound center. We then investigated whether these peptides could reverse VRI-induced inhibition of HUVEC migration (Figures 3B,D). Compared with the untreated control group, VRI significantly inhibited HUVEC migration at both 6 h and 12 h. This inhibitory effect of VRI was reversed by peptides in a time-dependent manner. At 6 h, only VEGF, MFDCLR and MDMWGK significantly attenuated the VRI-induced inhibition of HUVEC migration. At 12 h, except for MPGLGR, the other four peptides significantly reversed this inhibitory effect.

Subsequently, we performed the tube formation assay based on Matrigel matrix. Consistent with migration results, the five peptides significantly improved tube formation, showing increased branch length, branch number, and junction count compared to the control group (Figure 3E). Moreover, as shown in Figure 3F, fluorescence microscopy revealed that complex and diverse well-branched tubes formed in the control group. However, only a few short branches began to form in the VRI-treated group. After treatment of VRI-injured HUVECs with the peptides, VEGF, MDMWGK, MNPGCR, and MPGLGR significantly enhanced tube formation and increased branch numbers, whereas the other two peptides (MFDCLR and MYDNFCK) showed no significant difference compared with the VRI group. Notably, MDMWGK exhibited the most significant pro-angiogenic activity, as reflected in its ability to reverse VRI-induced migration inhibition as early as 6 h and to induce the most robust tube formation. Based on these results, MDMWGK was selected for subsequent mechanistic investigation.

### 3.5 Effects of five selected peptides on gene expression and protein secretion in HUVECs

**FIGURE 4:**
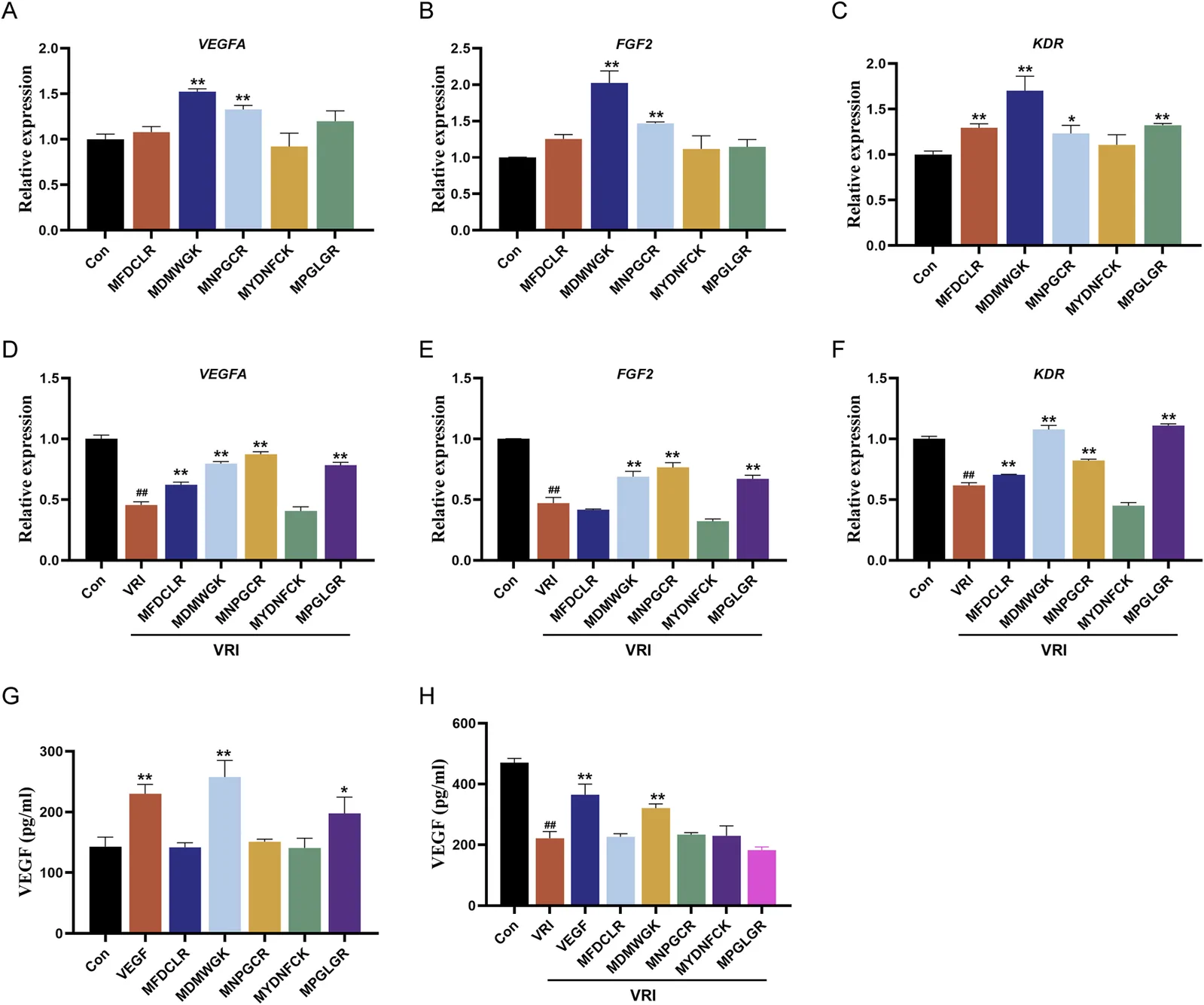
Effects of five peptides on angiogenesis-related gene expression and VEGF protein secretion in HUVECs **(A–C)** qRT-PCR analysis of the relative mRNA levels of **(A)** VEGFA **(B)** FGF2, and **(C)** KDR in HUVECs treated with five different peptides alone. Values are shown as means ± SD (n = 3). ^*^ *P* < 0.05, ^**^ *P* < 0.01 vs. Control group **(D–F)** qRT-PCR analysis of the relative mRNA levels of **(D)** VEGFA **(E)** FGF2, and **(F)** KDR in HUVECs treated with five different peptides and VRI. Values are shown as means ± SD (n = 3). ^*##*^ *P* < 0.01 vs. Control group, ^**^ *P* < 0.01 vs. VRI group **(G)** VEGF protein secretion in HUVECs after treatment with peptides alone was measured by ELISA. Values are shown as means ± SD (n = 3). ^*^ *P* < 0.05, ^**^ *P* < 0.01 vs. Control group **(H)** VEGF protein secretion in HUVECs after treatment with peptide and VRI was measured by ELISA. Values are shown as means ± SD (n = 3). ^*##*^ *P* < 0.01 vs. Control group, ^**^ *P* < 0.01 vs. VRI group.

To further delineate the pro-angiogenic potential of the five candidate peptides (MFDCLR, MDMWGK, MNPGCR, MYDNFCK, and MPGLGR) at the molecular level, we used RT-qPCR and ELISA to detect the expression of angiogenesis-related factors in HUVECs. As shown in Figures 4A–C, treatment with the five peptides alone upregulated the mRNA levels of VEGFA, FGF2, and KDR to varying degrees. Specifically, MDMWGK and MNPGCR significantly increased the expression of VEGFA and FGF2 compared to the control group. For KDR gene expression, MFDCLR, MDMWGK, MNPGCR, and MPGLGR all showed significant upregulation. At the protein level, VEGF secretion was significantly enhanced by MDMWGK and MPGLGR, with MDMWGK exhibiting the strongest effect (Figure 4G). We also tested whether these peptides could rescue VRI-induced inhibition of gene expression. VRI treatment significantly reduced the mRNA levels of VEGFA, FGF2, and KDR (Figures 4D–F) and VEGF protein secretion (Figure 4H). Co-treatment with each of MFDCLR, MDMWGK, MNPGCR, and MPGLGR significantly reversed the downregulation of VEGFA and KDR as compared with the VRI group. For FGF2 gene expression, MDMWGK, MNPGCR, and MPGLGR exhibited significant restorative effects. At the protein level, MDMWGK significantly enhanced VEGF secretion compared to the VRI group.

### 3.6 Effect of MDMWGK peptide on blood vessel formation in mice

**FIGURE 5:**
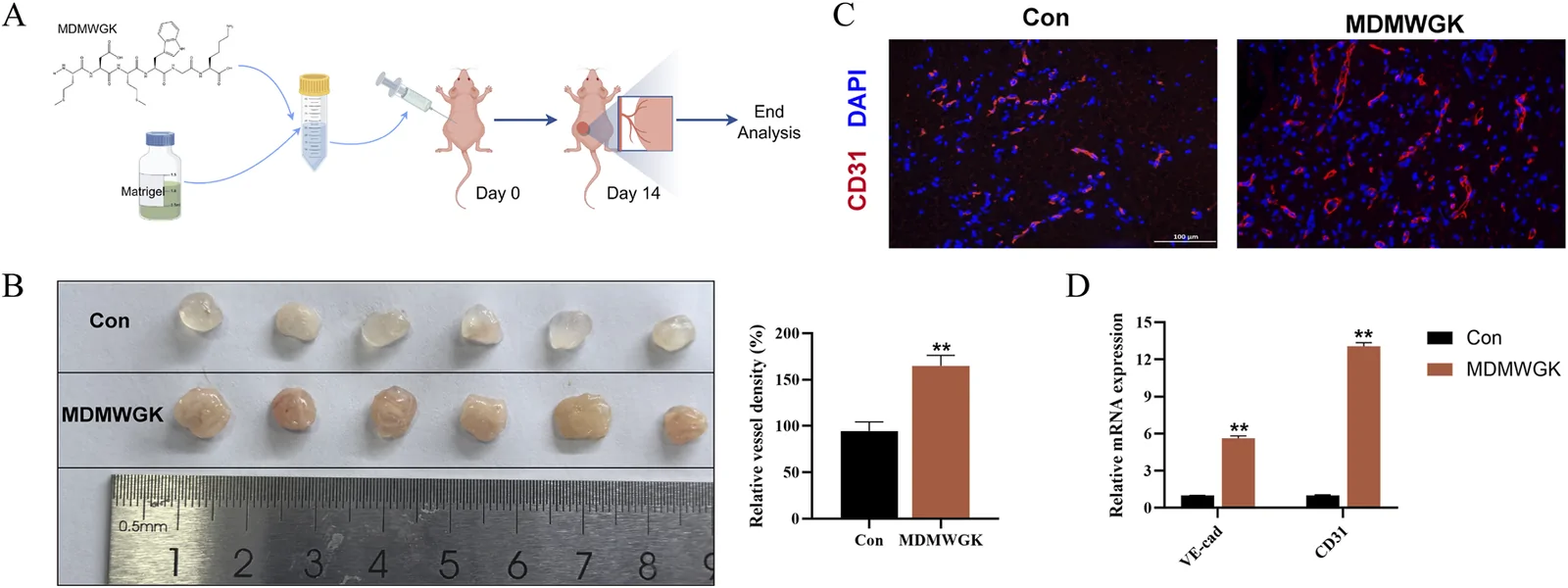
MDMWGK peptide promotes blood vessel formation in mice **(A)** Experimental scheme for **(B–D) (B)** Images of the Matrigel plugs retrieved from mice treated with or without MDMWGK peptide **(C)** Immunofluorescence staining and quantification of vessel density of CD31^+^ vessels in the Matrigel plugs **(D)** Quantification of mRNA expression levels of CD31 and vascular endothelial-cadherin (VE-cadherin) assessed using qPCR. Values are shown as means ± SD (n = 3). ^**^ *P* < 0.01 vs. Control group.

To further confirm the pro-angiogenic effects of MDMWGK peptide *in vivo*, we employed a subcutaneous Matrigel plug angiogenesis assay. The experimental scheme for this assay is shown in Figure 5A. As shown in Figure 5B, the matrigel plug in the control group remained transparent with no visible new blood vessel formation, whereas the plugs in the MDMWGK peptide group appeared light red, with evident angiogenesis visible to the naked eye. The mRNA levels of EC markers CD31 and vascular endothelial-cadherin (VE-cadherin) and immunofluorescence staining of CD31^+^ ECs also confirmed the effect of MDMWGK peptide on angiogenesis (Figures 5C,D).

### 3.7 Transcriptome analysis of ameliorating effects of MDMWGK peptide on VRI-induced vascular injury in HUVECs

**FIGURE 6:**
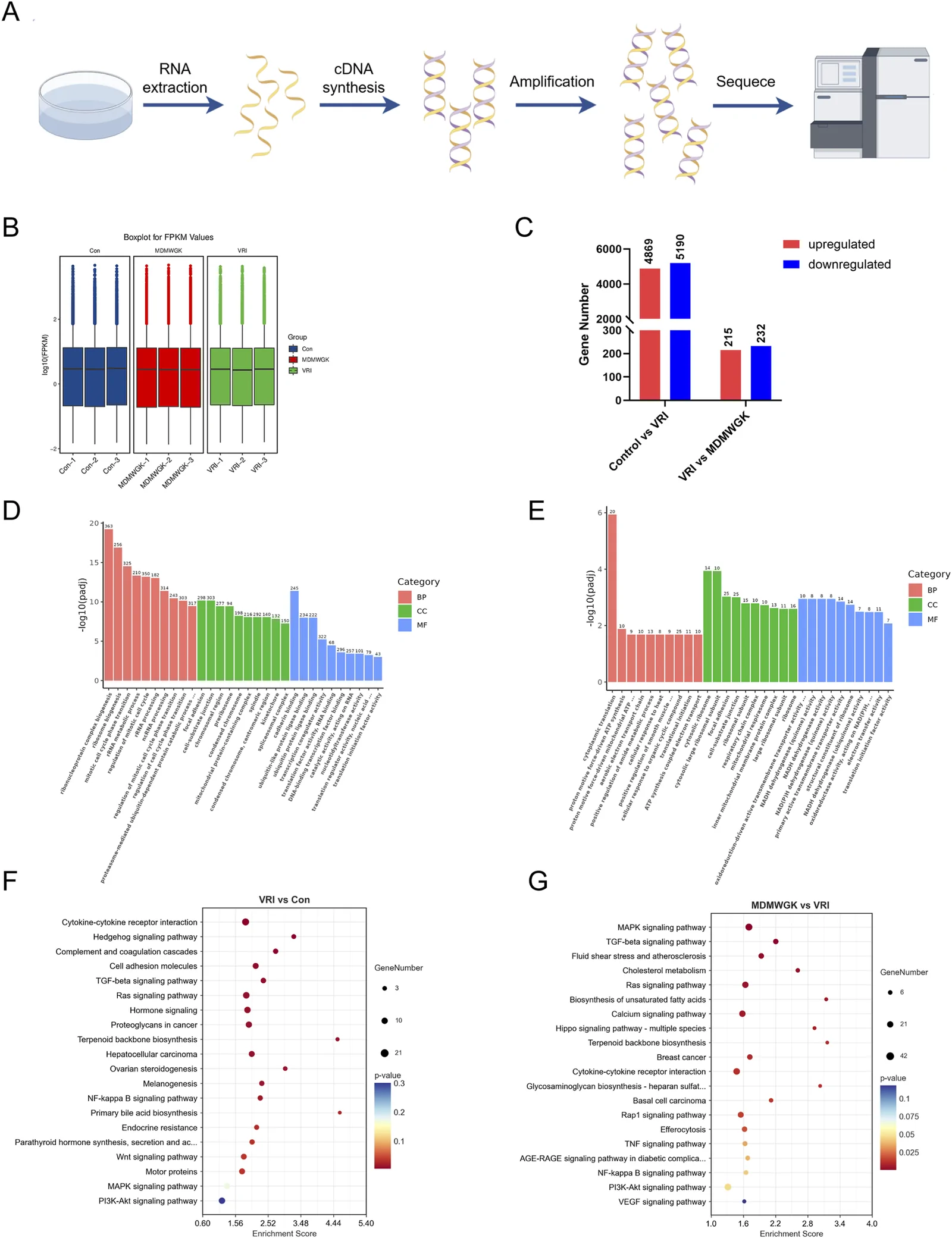
Transcriptomics analysis of the pro-angiogenic mechanism of MDMWGK peptide in VRI-induced HUVECs **(A)** Transcriptomics workflow diagram **(B)** The boxplots of gene expression (FPKM) in each group **(C)** Bar chart of the number of differentially expressed genes (DEGs) **(D)** GO term enrichment analysis of DEGs between VRI and control group **(E)** GO term enrichment analysis of DEGs between MDMWGK peptide and VRI group **(F)** KEGG pathway enrichment analysis of DEGs between VRI and control group **(G)** KEGG pathway enrichment analysis of DEGs between MDMWGK peptide and VRI group.

To determine the potential mechanism by which MDMWGK peptide protects HUVECs from VRI-induced vascular injury, we performed transcriptome analysis on the control, VRI, and VRI + MDMWGK groups (Figure 6A). The relative expression levels of genes under different conditions were quantified as fragments per kilobase of transcript per million mapped reads (FPKM). As shown in Figure 6B, the overall expression patterns of the three groups of genes were similar. Therefore, the study further compared the differentially expressed genes (DEGs) among the three groups using fold change (≥1.5) and P value (≤0.05) as criteria. By comparing with the control group, 4869 upregulated and 5190 downregulated DEGs were identified in the VRI group. Alternatively, compared to VRI group, those in the MDMWGK peptide group had 215 upregulated and 232 downregulated DEGs at concentration of 10 μM (Figure 6C).

Next, we performed GO enrichment and KEGG pathway analyses of the DEGs.The GO analysis results were ranked by enrichment score, and the top 30 annotations across biological processes (BP), cellular components (CC), and molecular functions (MF) are presented as histograms (Figures 6D,E). Subsequently, according to the results of the KEGG analysis, the transforming growth factor β (TGF-β), nuclear factor kappa B (NF-κB), mitogen-activated protein kinase (MAPK), and phosphoinositide 3-kinase-protein kinase B (PI3K-Akt) signaling pathways were enriched in the VRI-treated group compared with the control group (Figure 6F). Similar results were also observed from KEGG analysis in the MDMWGK peptide-treated group compared with the VRI group (Figure 6G). Among these signaling pathways, the PI3K-Akt pathway has attracted much attention due to its central role in promoting angiogenesis and cell survival (Chen et al., 2019; Li et al., 2025). Notably, the VEGF/VEGF receptor axis acts upstream of PI3K-Akt to regulate endothelial function and angiogenesis (Jiang et al., 2020). Given this regulatory relationship, we hypothesize that the pro-angiogenic effects of the MDMWGK peptide are mediated by these two signaling pathways.

### 3.8 Effect of MDMWGK on PI3K/akt/VEGF signaling in VRI-injured HUVECs

**FIGURE 7:**
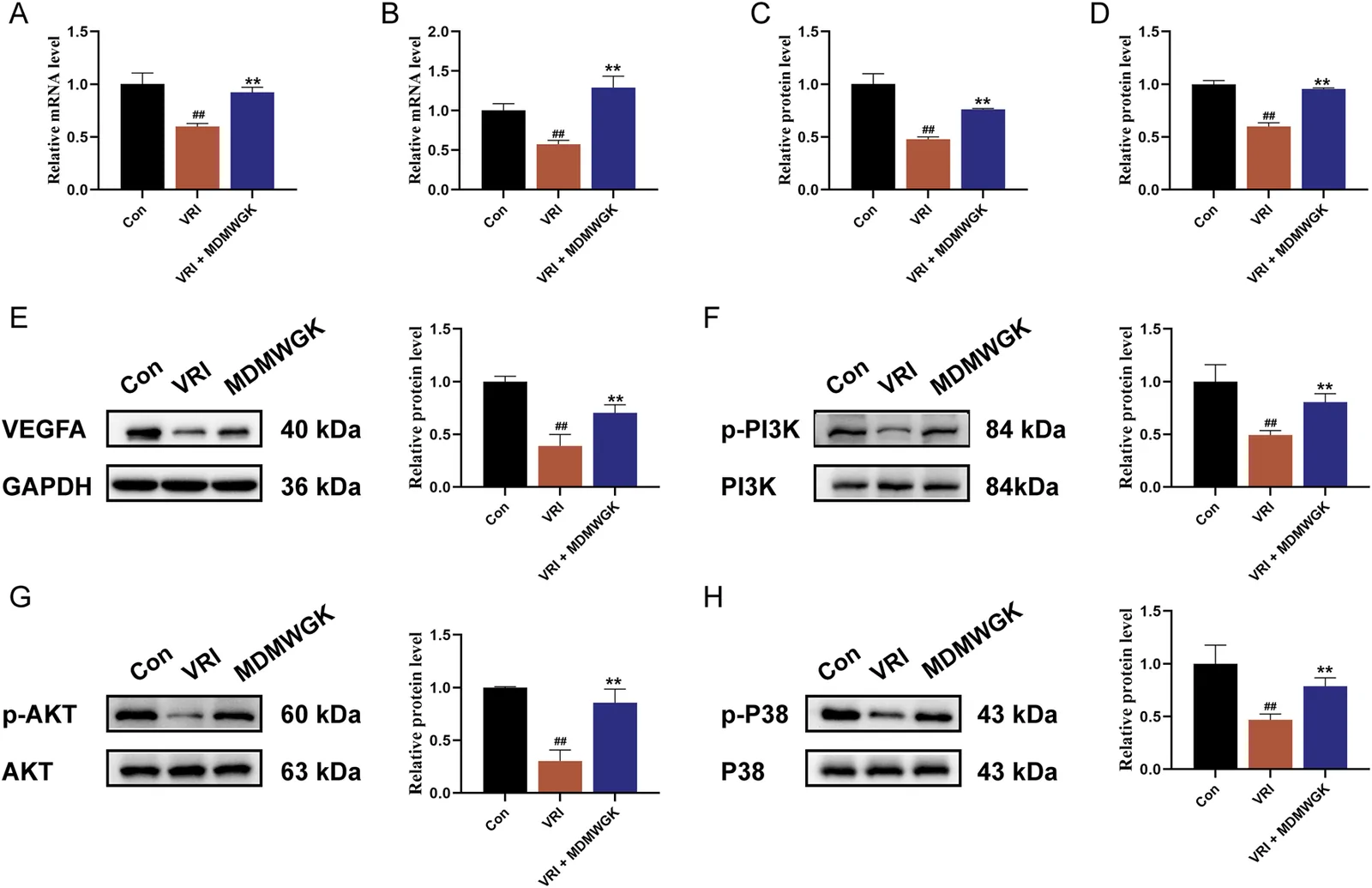
Effects of MDMWGK peptide on PI3K/Akt signaling pathway in VRI-induced HUVECs **(A-D)** The mRNA expression levels of VEGFA, VEGFR1, PI3K, and Akt in HUVECs. Values are shown as means ± SD (n = 3). ^*##*^ *P* < 0.01 vs. Control group, ^**^ *P* < 0.01 vs. VRI group **(E-H)** Effect of MDMWGK peptide on protein expression of VEGFA, p-PI3K, p-Akt, and p-P38 in HUVECs. Values are shown as means ± SD (n = 3). ^*##*^ *P* < 0.01 vs. Control group, ^**^ *P* < 0.01 vs. VRI group.

To further validate the involvement of VEGFA and its downstream signaling pathways, qRT-PCR and Western blotting were used to detect mRNA and protein levels, respectively. As shown in Figures 7A–D, the mRNA expression levels of VEGFA, VEGFR1, PI3K, and Akt were significantly decreased in the VRI model group compared with the control group. However, MDMWGK peptide treatment significantly increased the expression levels of these genes compared to the VRI group. The Western blot results indicated that the protein levels of VEGFA, PI3K, p-PI3K, Akt, p-Akt, p38, and p-p38 in the VRI model group were significantly downregulated. Nevertheless, peptide treatment markedly increased the level of VEGFA expression as well as the phosphorylation of PI3K, Akt, and P38 in its downstream signaling pathway (Figures 7E–H).

## 4 Discussion

In this study, we successfully identified multiple novel pro-angiogenic peptides from *Spirulina* through an integrated strategy combining LC-MS/MS, bioinformatics, molecular docking, and zebrafish-based screening. Among the 20 synthesized candidate peptides, peptides MFDCLR, MDMWGK, MNPGCR, MYDNFCK, and MPGLGR exhibited pro-angiogenic activity in the Vatalanib (PTK787)-induced zebrafish vascular injury model. In particular, the peptide MDMWGK demonstrated the most potent angiogenic capacity among the five peptides in both *in vitro* and *in vivo* assays. Mechanistically, MDMWGK exerts its pro-angiogenic effects through activation of the VEGFA/PI3K-Akt signaling pathway. These findings provide a rationale for the development of novel therapeutic strategies to promote angiogenesis in ischemic diseases.

Bioinformatics and molecular docking techniques have potential applications in the evaluation, exploration, and efficient screening of candidate bioactive peptides from proteins (Hong et al., 2023). In this study, a total of 114 peptide sequences were identified from fraction B by LC-MS/MS, 41 of which were predicted to be potentially bioactive by PeptideRanker. To prioritize candidates with pro-angiogenic potential, we performed molecular docking of the 41 peptides with EGFR, FGFR1, VEGFR1, VEGFR2, and Akt. According to docking results, the top 20 candidate peptides with the lowest average LibDock scores were selected for subsequent synthesis and pro-angiogenic activity evaluation. Notably, peptides with lower molecular weights (<1 kDa) tended to exhibit higher levels of pro-angiogenic activity. This was particularly evident for peptides composed of fewer than ten amino acid residues, which demonstrate greater physiological activity owing to their favorable oral bioavailability (Lv et al., 2024).

Zebrafish embryos have been valuable models for real-time studies of vasculogenesis and angiogenesis due to their optical transparency and the availability of transgenic lines that allow *in vivo* visualization of cellular behaviour (Yu et al., 2016). In this study, we used the transgenic Tg (fli1a:EGFP) zebrafish line, in which endothelial cells specifically express Enhanced Green Fluorescent Protein (EGFP) under the control of the fli1 promoter, resulting in green fluorescence. This enables real-time visualization of vascular structures, particularly intersegmental vessels (ISVs) and subintestinal vessel plexus (SIVP) (Schuermann et al., 2014). To further screen pro-angiogenic candidate peptides, we used the VEGFR2/KDR inhibitor PTK787 to block VEGFR2 signaling and thereby inhibit angiogenesis. Consistent with previous reports (Liu et al., 2023), PTK787 treatment significantly shortened ISV length, mimicking impaired angiogenesis. Our results demonstrated that treatment with five selected peptides, especially MFDCLR, MDMWGK, MNPGCR, MYDNFCK, and MPGLGR, markedly restored PTK787-impaired ISV formation and promoted SIV sprouting.

HUVECs are commonly used as a cell model for angiogenesis studies. Angiogenesis is a complex process, and vascular endothelial cell proliferation, migration, and invasion are critical steps in angiogenesis (Roskoski, 2025). To establish an *in vitro* model of impaired angiogenesis, we employed the VEGFR tyrosine kinase inhibitor II (VRI), a specific VEGF receptor inhibitor that has been widely used to induce endothelial cell injury (Li et al., 2014). In this research, we established a HUVEC injury model by using VRI to inhibit the physiological functions of VEGFR. Peptides MFDCLR, MDMWGK, MNPGCR, MYDNFCK, and MPGLGR promoted the proliferation and migration of HUVECs, increasing the number and length of HUVEC tubules. Among them, MDMWGK exhibited the most pronounced pro-angiogenic effect. VEGFA, a well-known pro-angiogenic factor, governs vasculogenesis and angiogenesis and maintains vascular stability in embryonic development, physiological and pathological conditions (Zhao et al., 2019). KDR (also named VEGFR2) is the primary receptor for VEGFA and the key molecule for VEGF signaling in angiogenesis. In our experiment, MDMWGK significantly upregulated the mRNA expression of VEGFA, FGF2, and KDR, and also enhanced VEGF protein secretion in HUVECs. Furthermore, it effectively reversed the suppression induced by VRI. The results demonstrated that MDMWGK could promote angiogenesis and vascular repair by activating VEGFA and VEGFR2. We further assessed the pro-angiogenic effect of MDMWGK using a Matrigel plug assay *in vivo*. The results revealed that peptide treatment enhanced neovessel formation. To further confirm the formation of new blood vessels, we examined the expression of endothelial cell-specific markers. Cluster of Differentiation 31 (CD31) is a transmembrane glycoprotein that plays a key role in inflammation, vascular endothelial cell (VEC) migration, and angiogenesis. As a specific marker expressed in the early stage of vascular development, CD31 is widely used to evaluate newly formed blood vessels. VE-cadherin is an important adherens junction protein that plays a crucial role in vascular integrity (Yang et al., 2022). Both are well-recognized endothelial cell markers (Chovatiya et al., 2023). RT-PCR further showed that the mRNA expression of CD31 and VE-cadherin was elevated after peptide treatment, providing molecular evidence that supports the observed *in vivo* pro-angiogenic effect.

Transcriptomics provides a powerful and cost-effective way to assess transcriptional levels under multiple conditions and has been widely used to elucidate the mechanism of drugs in experimental models (Bian et al., 2024). Here, we performed transcriptome analysis on HUVECs treated with the MDMWGK peptide to investigate the mechanism underlying its pro-angiogenic effect. The results suggest that MDMWGK-mediated angiogenesis may involve VEGF, PI3K-Akt, and MAPK signaling pathways. The PI3K-Akt pathway is considered crucial for VEGF/VEGFR2-stimulated angiogenesis, mediating endothelial cell proliferation, migration, and tube formation. Akt, a downstream signaling intermediate of PI3K, plays a significant role in the VEGF-regulated angiogenic pathway. For example, the Ac-Tβ_1-17_ peptide can significantly promote the proliferation, migration, invasion, and tube formation of HUVECs through the upregulation of PI3K and Akt phosphorylation, as well as the increased expression of VEGFA (Rahaman et al., 2025). Therefore, Western blot experiments were conducted to verify the involvement of VEGFA and its downstream signaling pathway. The results showed that MDMWGK treatment significantly upregulated VEGFA expression and further promoted the phosphorylation of key proteins in its downstream signaling pathway, including PI3K, Akt, and p38. These findings suggest that MDMWGK promotes angiogenesis by activating the VEGFA/PI3K-Akt signaling pathway. However, it should be noted that while our data demonstrate a clear correlation between pathway activation and the observed pro-angiogenic effects, they do not establish a direct causal relationship. Future work using PI3K or Akt inhibitors will be required to determine whether activation of this pathway is necessary for the pro-angiogenic activity of MDMWGK.

## 5 Conclusion

In summary, this study successfully identified and characterized multiple novel pro-angiogenic peptides from *Spirulina* through an integrated strategy combining LC-MS/MS identification, bioinformatics prediction, molecular docking, and *in vivo* zebrafish screening. Among these, the peptide MDMWGK demonstrated the most potent pro-angiogenic effect. Transcriptomic analysis revealed that MDMWGK promotes angiogenesis and that this process is associated with the VEGFA/PI3K-Akt signaling pathway. These findings establish a robust screening strategy for discovering bioactive peptides from algal sources and provide a theoretical basis for developing food-derived pro-angiogenic agents, with MDMWGK representing a promising lead compound for ischemic disease therapy.

## Data Availability

The original contributions presented in the study are publicly available. This data can be found here: https://www.ncbi.nlm.nih.gov/sra/PRJNA1512430.
