# Supplementary material for: Screening and characterization of pro-angiogenic peptides from Spirulina: computer simulation, experimental validation, and mechanistic insights
Source: Front Pharmacol. 2026 Aug 25;17:1885188. doi: 10.3389/fphar.2026.1885188 (PMC13549824; doi:10.3389/fphar.2026.1885188)
Supplement: Supplementary file 1 [file DataSheet1.pdf]

Screening and characterization of pro-angiogenic peptides from *Spirulina*: Computer simulation, experimental validation, and mechanistic insights

Fenghua Xu [1], Bing Li [3], Shanshan Zhang [4], Yun Zhang [4], Guohai Su [1,2,*], and Yan Zheng [1,2,*]

[1] Research Center of Translational Medicine, Jinan Central Hospital Affiliated to Shandong First Medical University, Jinan, 250013, Shandong Province, China

[2] Department of Cardiology, Jinan Central Hospital Affiliated to Shandong First Medical University, Jinan, 250013, Shandong Province, China

[3] Department of Genetics and Cell Biology, Basic Medical College, Qingdao University, Qingdao, 266000, Shandong Province, China

[4] Biology Institute, Qilu University of Technology (Shandong Academy of Sciences), Jinan, 250103, Shandong Province, China

Supplementary Figures

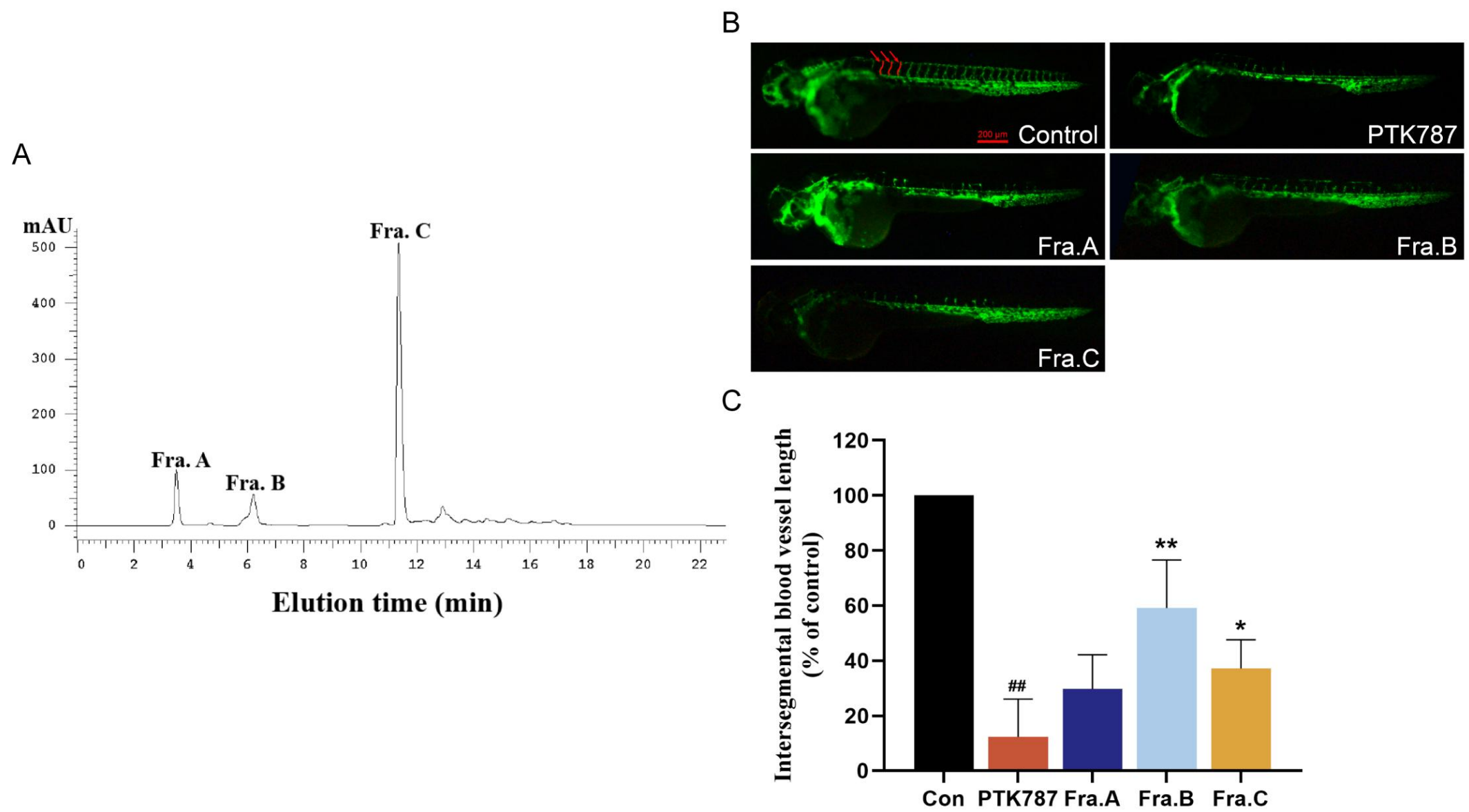

Fig. S1. Separation of *Spirulina* extract hydrolysate and pro-angiogenic activity of the purified fractions. (A) Purification of the hydrolysate by reversed-phase high-performance liquid chromatography (RP-HPLC). **(B)** Zebrafish embryos at 24 h post-fertilization (hpf) were treated with PTK787 alone or together with different fractions for 24 h. Images were captured at 48 hpf. **(C)** Quantitative analysis of the restorative effects of different fractions on PTK787-induced ISV impairment. Values are shown as means ± SD (n = 8). $^{##}P < 0.01$ vs. control group. $^{*}P < 0.05$ vs. model group, $^{**}P < 0.01$ vs. model group.

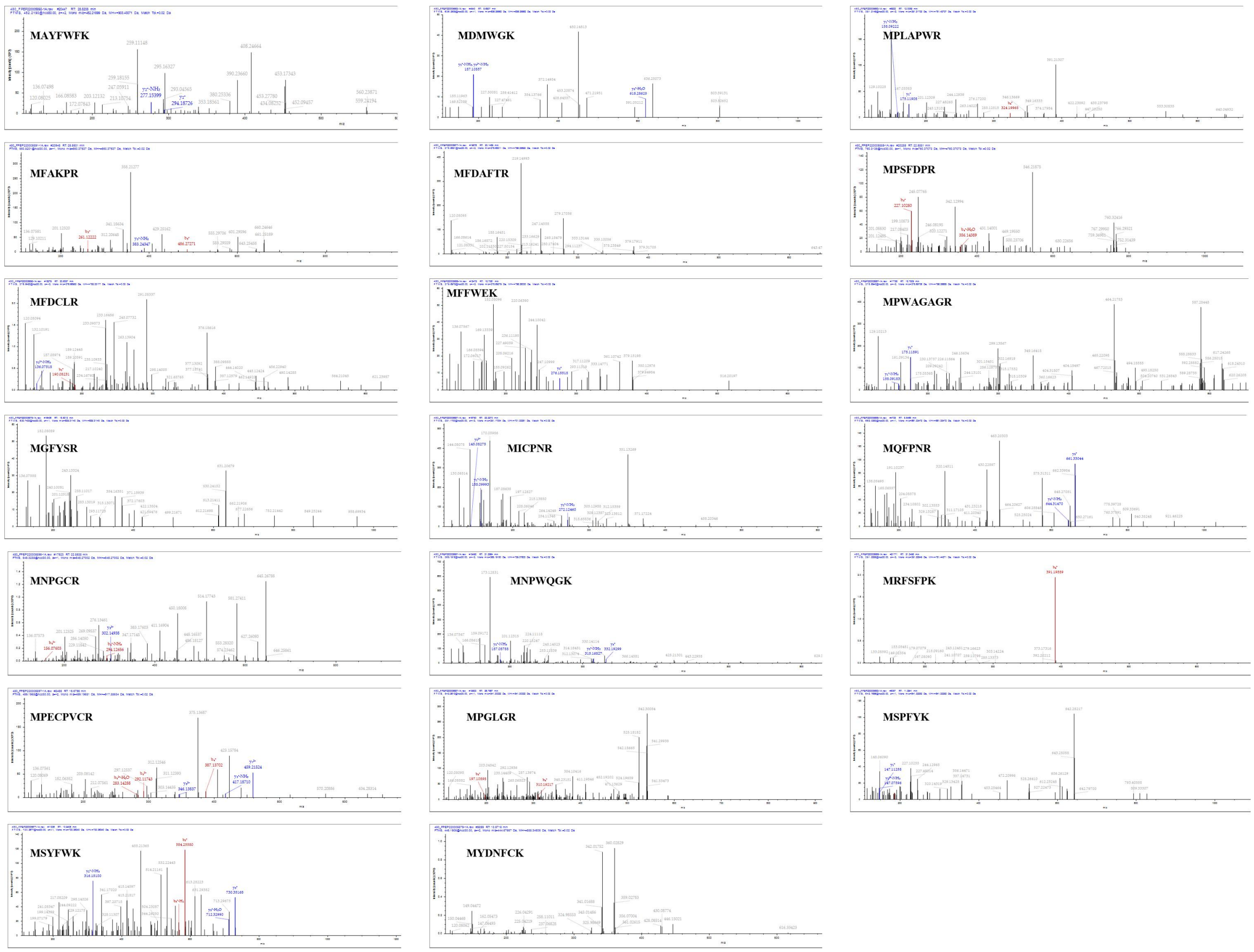

Fig. S2. MS/MS spectra and structures of the 20 peptides identified from the *Spirulina*.

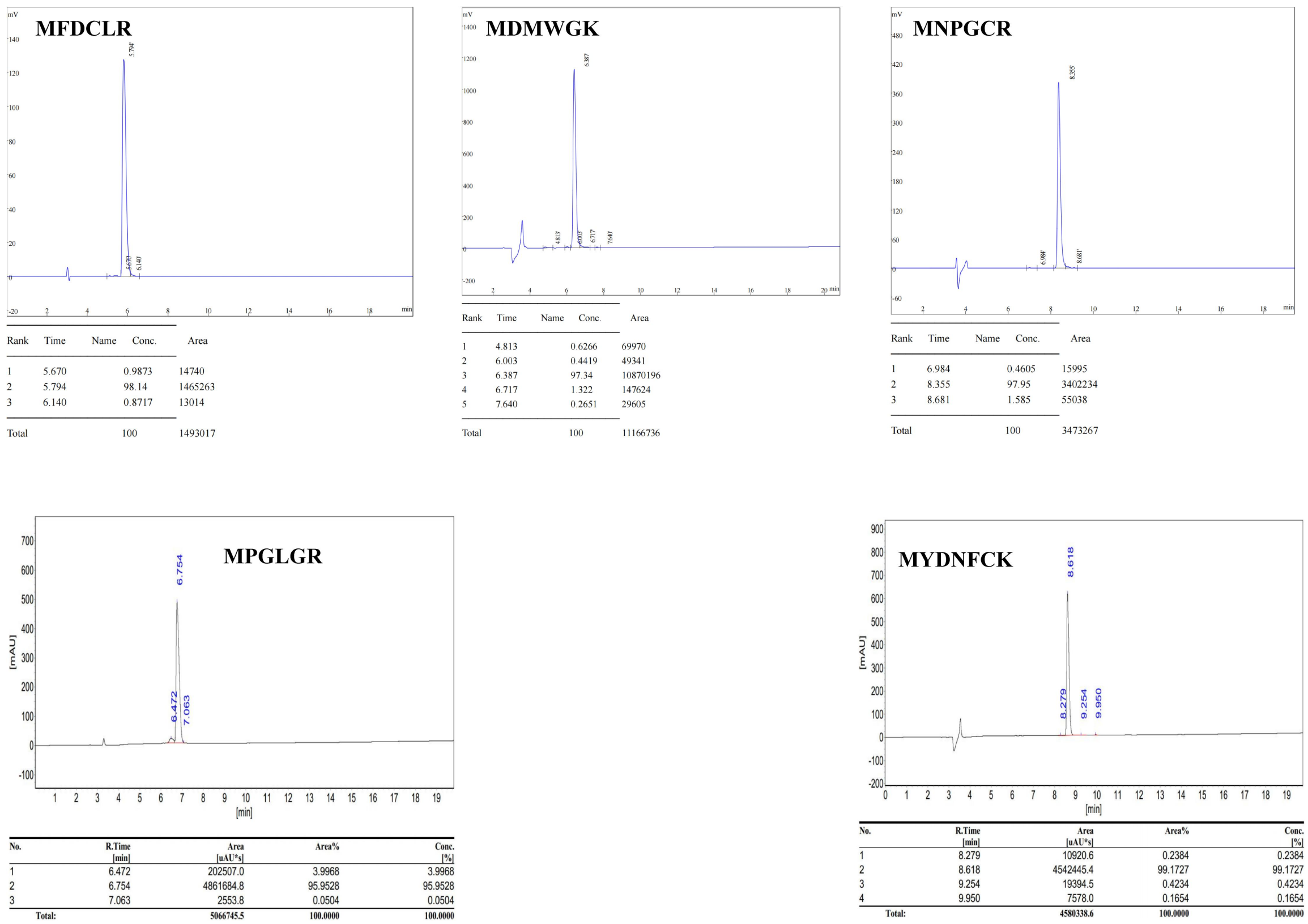

Fig. S3. Analytical RP-HPLC chromatograms of the 5 synthesized peptides

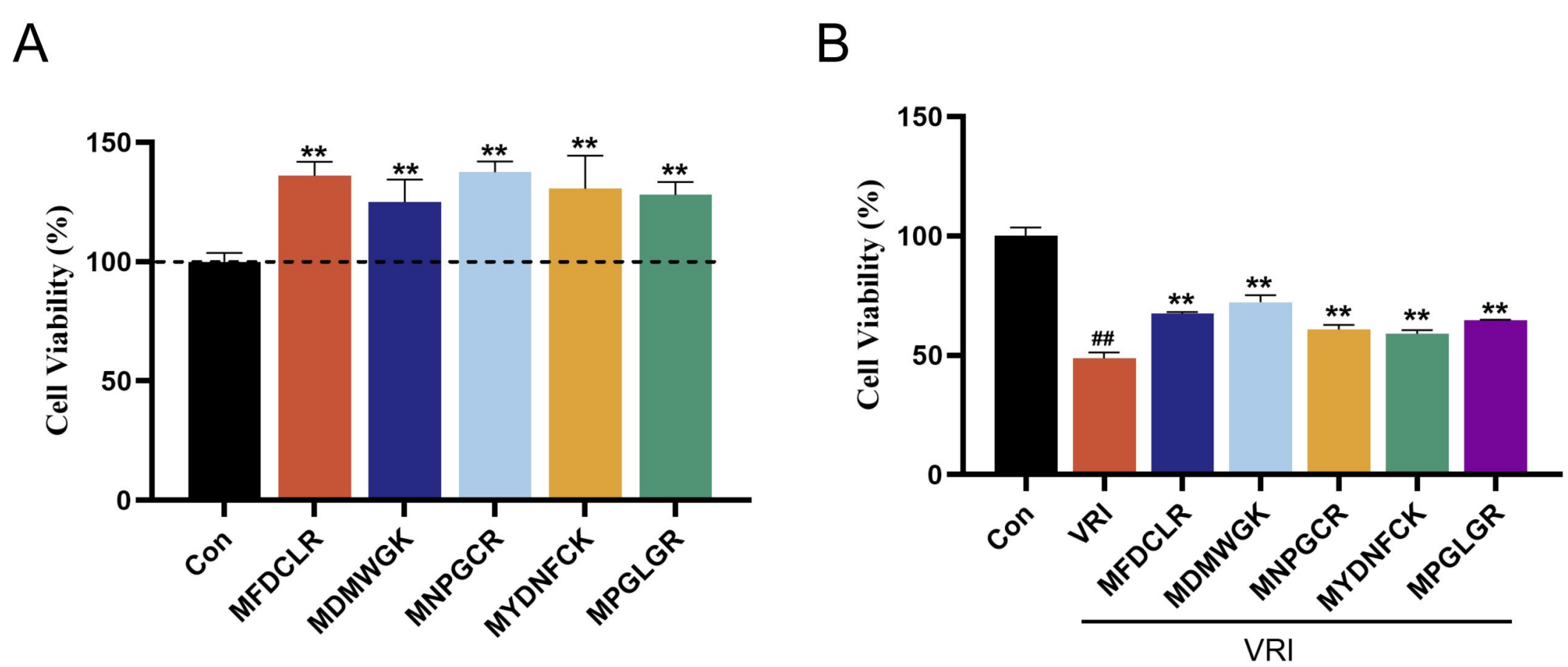

Fig. S4. Effects of different peptides on HUVEC viability. **(A)** Cell viability was measured by CCK-8 assay after treatment with different peptides. Values are shown as means ± SD (n = 3). $^{**}P < 0.01$ vs Control group. **(B)** Cell viability was measured by CCK-8 assay after treatment with VRI and different peptides. Values are shown as means ± SD (n = 3). $^{\#\#}P < 0.01$ vs Control group, $^{**}P < 0.01$ vs VRI group.

Supplementary Tables

Table 1. Sequence information and UniProt identifiers for *Spirulina*

Peptide sequence	Accession	Protein	Organism	Length	Sequence
MFDAFTR	A0A845WZN0	C-phycocyanin beta subunit	Spirulina sp. SIO3F2	172AA	MFDAFTRVVSQADARGAFLSTEQLDALQAIVAD<br>SNKRCDAVNRITSNASAIVTGAARALFAEQPQLI<br>QPGGNAYTSRRMAACLRDMEIILRYVTYAVLAG<br>DSSVLEDRCLNGLRETYQALGTPGASVAIGVGK<br>MKDAALAVANSPAGITPGDCSQLMAEIAGYFDR<br>AAAAVA
MFDCLR	A0A845X2J6	Terpene synthase	Spirulina sp. SIO3F2	375AA	MFDCLRSLEKTICLFPDQDDHPIISVEEFNLLISNI<br>NQVRGFELPLLSSSEQEFSSRFFYVNRFERSAMD<br>EIGSFYHYAHRSAGKLLSKKRIMELGLLIKFLSTE<br>PSTKIRSEIISDLILIYLTIIVFVQDFPGTHKHGLPY<br>RQKCDAIVNYLLEEKTIIELKTDIKSIATQTPLFDS<br>KFAEETSANSLIDKLVELLEQLHQKLETAQQQKL<br>ISQDLKRLFCQEFGKSIRTCHLPSIHTRAQLSGHL<br>AEASASCMLKPVFILLVSSLDVKTTHLEKWKYCI<br>EIVHLCMRLVNDFFSYEREKNTGTDHFRNLAHIL<br>QNERTNELQARSMVVQLYNLLMAHYLTAKNQP<br>LSEPEKELFSVLELVHDIPLFVWSELAA
MAYFWFK	A0A845X0I9	Protoporphyrinogen IX oxidase	Spirulina sp. SIO3F2	188AA	MAYFWFKSFHILGFVAWFAGLFYLPRLFVYHIE<br>AGERTAAEQAILNPEYSRMEMRLYKLIMTPAMI<br>FTALMAIGVIWTEPYVLKETWLHVKLLLVVILIG<br>FHFYCGRVRRQLEAGTCTITSIQMRRINEIPTVLL<br>GLVTLLAIFKNNLPTSTTAIGTGVAIVAFAVIIQL<br>YAKKRRLQKQQEELGTSG
MSYFWK	A0A845X7T9	ABC transporter substrate-binding protein	Spirulina sp. SIO3F2	385AA	MSYFWKRWWLVIACLTCCITVWACRPNPDPEIV<br>IGVIVPLTGDVSLTSGRPTLEGIELAVQMINEEGG<br>FEIKEQRYPVELIVADDQDDPSLAAAAAHQLINQ<br>DKVVALVGMPLSRNAIPVAQVAEAAGIPMISSK<br>STNPQTTAGKEYVFRVTFTDAFQAEVIATLVRD<br>KLQVQRAAVLYDVASDYNKYLAESFQTAFENR<br>GGDVVAFETYVTDNPQFEQSLEAIKLAQPEVLFL<br>PNYPAEVLNQAQMARDLGLDIPLIGADSWTGFG<br>DFSDPALEGALYTADWSDDIDTPQTTAFLAAYR<br>QQYNHVPTSAAALGYDAIQLLFTAIQTQDSFEPQ<br>MIQTGLTQIRDYPGVTGSISYGQGGDPSKDAAIL<br>QVQKGKSVFFESVRPKE

MDMWGK	A0A845X0U7	Uncharacterized protein	Spirulina sp. SIO3F2	213AA	MDMWGKWAKASLKYGLAGVVSLALVLWGLDGEHFVAAQSQSTEPTNAPTAACAQAAQGNRNIRAAEQAYGQLSDFPRENTYRLADSNKVQDCNTLLYRLIYYHTRTKRRSPSLRFDWKLTLGDYLGAHETIIPDRYPTATSLDENPLERDRSILDQLNRQQRNVLVNTLVVIFEGQIPSSAPDEKPVEVTPVPEPLQPETPATPQPGDSRLLL
MNPGCR	A0A845WYU1	Secreted protein	Spirulina sp. SIO3F2	70AA	MNPGCRSTALRVALCVGSVLFVINHGSALISGQMSRDRWISAMLTYCVPFGVSLHGQHQG
MFFWEK	A0A845X975	Translation initiation factor beta propellor-like	Spirulina sp. SIO3F2	449AA	MFFWEKGKPQKYWCFPTALLCTWIFSASGSNSIYNHDFIRSNSFAEEVSQNEFGDLSQKVRGFLAFDNKEDFHFVEWSSSGHYLIVENKSLEIVEVIRIRDKVSLYSVPYGEKFVSTVVNLSPNEKYLAIALKAKQSESLQKRYYLDILDLEVGQKIASFEHEGSIGDLHWSPDSQNIATVGLERGRGDNIVRAFSVGSNRELFQIAHNRGPRNAVNSFRWSPDSKYIATASRNGNTHIFSVSAHSEIMTIFHNQPVLDINWSPNSDFISTISLGGTKVVNILTQEELSGFGENSDEAFWSPDGKYISTDSFHDSRITNYSFVDMQEVSSIEYERMNYVPSQWSPDSRYIAAVESNNSVAIMNIENAQNIISIEHKGIVSDIAWSFDSRYLATVYDGKIKVFDTVSSRELKIPEINSRSEFVYWNPSGQYLVVEGFSPKPTVTVLLVHE
MQFPNR	A0A845WV92	DUF928 domain-containing protein	Spirulina sp. SIO3F2	378AA	MQFPNRRLFSQPLGLLLLASSLLSPSLLTHHAQALTPDGEGSRAQVASFQDADWNLIPYPDQNAPTQSRGGASRSGEWNLISYPDQGAPTQSRGGASRNGEWNLIPYPDQGTPSQSRGGASRNGEWNLIPYPDQGTPSQSRGGASRNGEWNLIPYPDQGTPSQSRGGTSRDGEWNLVPYPDQGIPAQSRGGSSRRTDLPQDMVTLMPEIGYGLTVSSHPTFFFYLPDSNAQAGLFRLKDAEGKVLYEQAIEQPNQAGIVAIALPEQLPELTIGDYYQWYVVLQSEGKFRPSSPFIDGWVQRVEPTPQVQQAEAGDNVLGYAQALTHQNVWYDTLTAINTIRTAQPHSVEAQQQWQALLTSVSLEALVDAPVVVQGTSH

MYDNFCK	A0A845X622	Rpn family recombination-promoting nuclease/putative transposase	Spirulina sp. SIO3F2	278AA	MYDNFCKYLIETYPDDFVAWLIGKPVALTRLEPTELISDPIRADSLLLQGQDVVLHVEFQTQPDKQIPERMANYYLRIRKKFATQHIVQVVVYLRATDSPLVQENVFQSGGMTHRFEVIRLWEQPKAVFWDAPGLLPFAILSKAANEDAAALLEQMRVRINEVTPDVAMQGNLAMATAVLAGLKLSPVVIQEVMRSKAMRESLFYQEILREGRAEGRQEGRQEGRVEGRQEGRLMMLRQVVQILQGWGFPVERLLKIVGITMAELEQVELSHEDEASS
MPECPVCR	A0A845X7E4	Chromosome segregation ATPase	Spirulina sp. SIO3F2	1006AA	MPECPVCRTRYVKGRLNRCHVCGWQFDSSGFSMIGPVKIQHQLSNVALGRIQAWAHHMWTTIQQQKERIVQLQTQLRQLQNQAAEQKALPASANAVASPPQLPTSAIEAPQPPTANASPEMWQWVLTRLEHAEQERERLKAQIAQLQAQPTQPPALQGTDVKPEMQTELIKWREESRALQYQQSEQWQTIFEHFRYNTQQALTQHEQKLDTTLEPLSTQLTSQEQAIEQLEHQLQDLQHHADGAMQGAIAPLTDQLQSLQTTQGEISQQFKHQLESFQQAQADWATQQEQTVQQTQQVLDAQEQQWQGAIDPLQHRLATQEQAIAALEARLHEARSQAIEQLTAQVQDIQNQSEAGLESAITPLAAQLQSLQSAQDEHRHRLESFQQTQSDWATEQEQKLQHTQTYWATQQEQKLNQEQAAWMMQQEQKLQQTQQVVAEQAQQLQDTLSPLQAQITAQTQAIAESETQLQAAQRQAVEELKVQIQGVQAHSETTLQGTIEPLRSQIEVQKQAIAALETQLQASQNQANEQLQAQLQAVQTQSEARVQGAVAPLAEQIQGLRIDQGARFGQLEQQCESLQQTQVDWATQQAQNLSDAIAPFNSQIEAQQQAIAELETQLQASQVQAISQFETQLQNFKNQSEATVQGAIDPLNRQIESQTQALTELETQLQAMQTQSEVVVQGAIEPLNKQLEAQKQASSELETRLQASQNQANEQLQAQLQAVQNQSEAKLQGAIAPLTEQIQSLKTDQGDRFSQLEQQCESLQQTQVDWTNQQAQNLSDVIAPLNSQIEAQQQALAALKTQLQTSQEQALAPLTTQLQSLEAAQRELATMPEQVRSLQTMVEQQQLQTPQLEQSIITLTTQFQTLEHSQDQSVHQLADQIVQLQHTQEQTAQLISPTEICATLERLQTQLQDLRTVQEQTLSLPQEIESLELMVQQHVQATEPASLAWQRQKNEILAKIDRFDTICEQFQTLESTVAQLQKAIHTLISPSTVPTRRTNT

MPLAPWR	A0A845X5C5	Pyridoxamine 5'-phosphate oxidase	Spirulina sp. SIO3F2	193AA	MPLAPWRSPLARALHRNRAAADARYCQLATVNADGYPSNRTVVFRGFWQESNHLQIITDARSTKLAHLQRQPWGAICWYFRQSREQFRLSGEINVIDHRTPRPDLQQARRQMWQNISDNARAQFLWPDPGVPKADPAAFEVVPDPATIPETFVLLWFNPEQVEHLELRGEPQNRTRYQYEHRTGQWQVEALNP
MFAKPR	A0A845XA59	DUF928 domain-containing protein	Spirulina sp. SIO3F2	226AA	MFAKPRSTPGSQDASGEPVTQTVGGGTRNDPLFPSENSNPTEDFQAPLPQPSAQAEPPLTPLVPQNNIILTQQERVNLYVYVPPTIVNKTAVLEIVDLNSQETVFMEFFEFESTETITRLVLPNTITFETTHPSDQRYQWRLYAYFAQHSTAQLLTEGWINRVEPSQESSSVLWHENLELWFAQREDSPTVWQQGLATETLERYADYPVQTYMFGVSEHSQAAGES
MICPNR	A0A845X4W3	Uncharacterized protein	Spirulina sp. SIO3F2	140AA	MICPNRLGEYGALQRCSDLMNDSRPDQDFDKAIDAAIAKHPHITDVDIIKTKHLITIKLQVDEKAAEQTEPEPTPPKTLKPMRSRSNPTALSPQELCQHYGLKKERVKTVAKNRDIAEWEALEQLTGWVWYPRKRVFRPK
MRFSFPK	A0A845X033	histidine kinase (Fragment)	Spirulina sp. SIO3F2	673AA	MRFSFPKIRSLFKISLQWVLIIPFLFQIFGTVGLVGYLSFQSGREAVETLAAEVINDTNDVVAEHLSSYLEMPQKLTQMNADAIRRGVLDLKDHERSAHYFWDQMQLYDLTYNGIGMIDGSGYGIARYDGQTATLEEWTGDTKDNIVTYALDAQGNPDEVLGVWTWSNFEESWYTNPINAGKPIWSIVETYLEQGGSVVFASASRPIYDPQGKLLGMLAADVQLLKLSDFLENLHIGQSGSVFIVERDGRLIANSGGISPFIVKPDHSTERFLASASPELLVQQVSQTIQEQFGGFHNLDQATELRLKFEGEPYFVSVHPWQEANGLDWLVAVAVPESEFLAEINANRSNTIALCLLALGAATGFGFATTRWIIHPILQLNQASSAIATGNLEQTVQGSPILELDGLTESFNYMAGQLRDSFTALEASKEQLEDRVNDRTAELKATLIELRNTQAQVIQNEKMSSLGQLVAGIAHEINNPVNFIYGNLSHTEAYVKDLLGLVKLYEQQYPEPNPAIEAEAEAIDWDFIQTDLPQMLTSMQTGTERIRAIVLSLRNFSRTDESALKTVDLHEGIDSTLMILKHRLKAQSERPAIEVVKNYGLTASIECYPGPLNQVFMNIISNAIDALEERAEQQTPEERECKPGIMTIRTRFVDDQWAEVAIADNGLGIPDDL

MPSFDPR	A0A845X4L4	CRISPR type III-B/RAMP module-associated protein Cmr5	Spirulina sp. SIO3F2	142AA	MPSFDPRTLNKYAFKALQKLSDDHKGKKARRKDQKNQAQELYTYISTWGLMRLKAEEKVLKSQEGRQEVVEIFFKKLKKLLGMEKDQKLDLNYLVGLPADQYLGLTGLAIELANEFAFWANAVYADLEGAQAPDPQETSKPI
MPGLGR	A0A845WYB0	N-(5'-phosphoribosyl)anthranilate isomerase	Spirulina sp. SIO3F2	227AA	MPGLGRTRVKVCCIASEEEARIAIRAGADALGLVGAMPSGPGVISDAIASKITKIVPPPIATFLLTSETTAQGIYEHVRLVRPTTVQVVNHIDRAEYEKLETILPRNTIKRVQVIHIESSESLNLINHYIPYVDAFLLDSGRPSAKVPELGGTGRVHDWNLSAEFVRRSSKPVFLAGGLRSENVEEAIKTVKPFGLDLCTGVRTDGRLDEGKLKAFMRVVYNHTVAQ
MSPFYK	A0A845WVF6	TIGR04372 family glycosyltransferase	Spirulina sp. SIO3F2	408AA	MSPFYKLLKLVLKYLYQFVWGYFFLYAIILIKPILLVRIGELESRAIGHFSLSVEIYLSELDRGIYDCKSRHIDLFFINRKKCNDVLVKKWKSFFRFLPALLLKPTFLLLLEFDSHREHLVPFRHWKIYSLASGYWQNCDIHDVLPKTRPHLKFTEQEHYAASQQAFSMGISPGDKLICFHARDPSYRSGSDAPFSFRDSSINLQVQAMLELAELGYKVVRVGKRVLEHLDCNHPNVIDYANTNIRSDLMDLYLISRCSFMVGTGSGIDAVASVFRIPVIYVNLADWGYLDQYSEWLWPLFIPKKLCWKSSGALLTFNEIYKLDLHCATHDSLYSEAGVDYLSNSPVEIADLVLEAEARLSGVWLDTQED
MNPWQGK	A0A845XB22	NfeD-like C-terminal domain-containing protein	Spirulina sp. SIO3F2	60AA	MNPWQGKATVYQTIHPNRPGRVLFNDTPWKARAQNGQTIPSGATVEVCDRQGIELIVKLF
MGFYSR	A0A845XBD6	Class I SAM-dependent methyltransferase	Spirulina sp. SIO3F2	203AA	MGFYSRLIVPAALDWVMSGSTFSEYRRSLLANISGTVLEIGVGTGLNLAHYPETVTHLTTIDVNPGMSRRAQARLAQLHCPTHHQVLNSETLPMGNNTFDHVVSTWTLCSIAQVSQALAEIYRVLKPGGQFHFIEHGLSDRPAVQIWQHRLTPLQKRIADGCHLDRPIQALIARQFATFTVEQFVMPDFPAVAGYTYKGVATK
MPWAGAGR	A0A845XDC9	Uncharacterized protein	Spirulina sp. SIO3F2	122AA	MPWAGAGRINLTLDWQATAIELGTSQYITITPEFTPSESLILPKVVWRVGYTSGGLTTYSETRSFWPQEHVGRVFQLPSPPPDGSDRHIVAKAIRPNFLRSLPDAEALARLEVIELDINKFE

Table 2. Primer sequences for HUVEC cell genes in qRT-PCR

**Primer**		**Sequence**	**Length**
GAPDH	Forward	GCACCGTCAAGGCTGAGAAC	20
	Reverse	TGGTGAAGACGCCAGTGGA	19
VEGFA	Forward	GCAGAATCATCACGAAGTGGT	21
	Reverse	CCAGGGTCTCGATTGGATGG	20
FGF2	Forward	AGAAGAGCGACCCTCACATC	20
	Reverse	CAGTTATCTGAAGAAAATGCAGAC	24
KDR	Forward	GGTTGTGTATGTCCCACCCC	20
	Reverse	GAGTGGTGCCGTACTGGTAG	20
VEGFR1	Forward	TGCCCGGGAGAGACTTAAAC	20
	Reverse	TGGCCAATGTGGGTCAAGAT	20
PI3K	Forward	TTGGAAGCAGCAACCGAAAC	20
	Reverse	CTTTACTTCGCCGTCCACCA	20
AKT	Forward	GGACAAGGACGGGCACATTA	20
	Reverse	CGACCGCACATCATCTCGTA	20

Table 3. Primer sequences for mice genes in qRT-PCR

Primer		Sequence	Length
GADPH	Forward	TGTCTCCTGCGACTTCAACA	20
	Reverse	GGTGGTCCAGGGTTTCTTACT	21
VE-cadherin	Forward	AAGACATCCGAGTGGGCAAG	20
	Reverse	CTGTACTCGCCCTGCATGAT	20
CD31	Forward	GCATCGGCAAAGTGGTCAAG	20
	Reverse	GGGTGCAGTTCCATTTTCGG	20
